# Engineering Osteoimmune Responses with Functionalized Orthopedic Implants for Post‐Operative Osteosarcoma Treatment

**DOI:** 10.1002/advs.202512363

**Published:** 2025-10-05

**Authors:** Yilong Dong, Lingshuang Wang, Hui Zhang, Menghuan Li, Zhong Luo, Yan Hu, Chunyuan Cai

**Affiliations:** ^1^ Ruian People's Hospital The Third Affiliated Hospital of Wenzhou Medical University Wenzhou 325200 China; ^2^ School of Life Sciences Chongqing University Chongqing 400044 China; ^3^ Department of Breast Chongqing Hospital of Traditional Chinese Medicine Chongqing 400021 China; ^4^ College of Bioengineering Chongqing University Chongqing 400044 China

**Keywords:** bone defect healing, orthopedic implants, osteoimmune system, osteosarcoma immunotherapy, post‐operative osteosarcoma treatment

## Abstract

Surgery is a mainstream treatment of osteosarcoma in the clinic, which often causes critical‐size bone defects due to its highly invasive nature. However, the post‐operative osteosarcoma management still remains challenging, characterized by high risks of recurrence and insufficient defect recovery. Recent studies increasingly reveal that the osteoimmune system is a crucial mediator of bone homeostasis and immune protection functions, emerging as a crucial nexus linking the antitumor immunity and osteopromotion regimes. Consequently, there is significant interest to develop new strategies to modulate the immune responses in the post‐operative defect site, aiming to sustainably inhibit residual tumor growth and recurrence while accelerating defect regeneration. Interestingly, orthopedic implants emerge as optimal platforms for the on‐demand engineering of immune responses in the post‐operative defects in a highly integrative approach. Herein, a comprehensive review is provided on the principles and designs of osteoimmunomodulatory orthopedic implants for evoking osteosarcoma‐specific immunity while boosting osseointegration and bone defect recovery. Overall, osteoimmunomodulatory orthopedic implants offer immense potential for ameliorating the osteosarcoma treatment outcome by improving patient survival and quality of life.

## Graphical abstract

Osteosarcoma is the most common primary bone tumor with limited treatment options and a terrible prognosis. This review provides a comprehensive summary of the recent development of osteoimmunomodulatory implants for post‐operative osteosarcoma treatment, of which the potential utility in evoking durable anti‐osteosarcoma immunity and accelerating bone defect healing is critically discussed, offering insights for improving osteosarcoma treatment outcomes in the clinic.

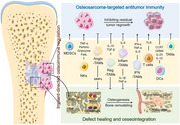

## 1 Introduction

Osteosarcoma is a malignant tumor that originated from the mesenchymal stem cells (MSCs) or committed osteoblast precursors, which is the most common bone tumor worldwide and affects people of all ages.^[^ ^1^, ^2^, ^3^, ^4^, ^5^ ^]^ From a pathological perspective, osteosarcoma is a highly aggressive disease featuring rapid growth and high metastasis potential to other bones and lungs, which often leads to severe systemic tumor burden with poor overall survival. Currently, surgery is the mainstream treatment option for osteosarcoma in the clinic, which ranges from conventional amputation surgeries to advanced limb‐preserving surgeries.^[^ ^6^, ^7^, ^8^, ^9^ ^]^ The patients usually receive neoadjuvant chemotherapy to shrink the primary tumors to increase the success rate of resection, while adjuvant chemotherapy is often applied to eliminate residual osteosarcoma cells in the post‐operative wound margin.^[^ ^10^, ^11^ ^]^ Nevertheless, the therapeutic outcome of these modalities remains limited. Indeed, despite extensive resection and combination chemotherapy, the prognosis of osteosarcoma patients is still largely unsatisfactory due to the high risk of post‐operative recurrence and metastasis.^[^ ^12^, ^13^, ^14^, ^15^, ^16^ ^]^ According to the results of the EURAMOS‐1 trial, the 3‐year event‐free survival estimate for patients with high‐grade osteosarcoma after standard neoadjuvant and adjuvant MAP therapy (combination of doxorubicin, cisplatin, and high‐dose methotrexate) was only 55%,^[^ ^17^ ^]^ while the 5‐year survival rate of patients with metastatic and recurrent osteosarcoma is below 30%.^[^ ^18^, ^19^, ^20^ ^]^ On the other hand, the surgical treatment of osteosarcoma may also elicit various short‐ and long‐term impacts on the patients that affect their quality of life (QoL). Particularly, the invasive nature of osteosarcoma often necessitates extensive resection of the tumor tissues and thus creates large‐size bone defects. Surgery‐induced bone defects cannot heal naturally under most circumstances as they exceed the natural bone regeneration capability, which not only increases the risk of post‐operative complications but also induces significant disability and functional impairment.^[^ ^21^, ^22^, ^23^ ^]^ The administration of neoadjuvant and adjuvant chemotherapy also impairs the regenerative capability of osteogenic cell populations at the wound margin due to their indiscriminate cytotoxicity, thus further hindering the reconstruction of the bone defect. Indeed, according to an analysis of 198 osteosarcoma patients, massive bone allografts for post‐operative bone reconstruction have an average survival rate of ≈60% for 10 years, which further decreased to ≈56% after 20 years.^[^ ^24^ ^]^ Notable post‐surgery complications in osteosarcoma patients include infection, mechanical failure, and non‐union. Consequently, new post‐surgery osteosarcoma treatment strategies are urgently needed with durable tumor inhibition function and robust osteopromotion capabilities, which are crucial for prolonging patient survival and improving their QoL in a clinical context.

Recent studies increasingly reveal that the bone‐residing immune cells intensely interact with the bone tissues at anatomical, cellular, and molecular levels, which are critically involved in various aspects of bone health, including osteogenesis, bone remodeling, injury responses, immune defense, etc.^[^ ^25^, ^26^, ^27^, ^28^, ^29^ ^]^ There is already concrete evidence that osteoimmune defects are linked to the pathogenesis and progression of various skeletal diseases.^[^ ^30^, ^31^, ^32^ ^]^ For instance, enhanced presence of myeloid cells in the bone microenvironment is associated with a higher risk of osteoporosis in postmenopausal women, while elevated natural killer cell frequency is correlated to poor responses to bisphosphonate treatment in osteoporosis patients.^[^ ^33^ ^]^ Alternatively, osteosarcoma cells could actively interact with the ambient tissue and immune cells to orchestrate a highly immunosuppressive microenvironment, of which the hallmarks include enhanced recruitment of regulatory T cells as well as severe exhaustion of infiltrating cytotoxic CD8+ T cells, leading to limited T cell clonality and tumoricidal potential for driving the immune escape of osteosarcoma cells.^[^ ^34^ ^]^ Indeed, the osteoimmune system is already widely recognized as a crucial nexus linking the antitumor immunity and osteopromotion regimes, highlighting the importance of regulating immune responses in the post‐operative bone defects for improving osteosarcoma patient outcomes.

The aggressive osteosarcoma surgery often results in significant challenges in the structural integrity and locomotion of the skeletal system in osteosarcoma patients. To facilitate post‐operative bone reconstruction and rehabilitation of osteosarcoma patients, various forms of orthopedic implants are applied to stabilize the damaged bone tissues and promote defect healing. Interestingly, owing to the rapid advances in material science and biomedical technology, the concept of orthopedic implants has shifted from mere fixative and supportive tools to multifunctional therapeutic platforms, offering facile solutions to overcome a variety of practical challenges in bone tumor treatment.^[^ ^35^, ^36^, ^37^, ^38^ ^]^ For instance, there are numerous reports on implant‐mediated in situ delivery of therapeutic substances, including osteogenic drugs, antitumor therapeutics, and antibiotics.^[^ ^39^, ^40^, ^41^, ^42^, ^43^ ^]^ Meanwhile, the structure and composition of orthopedic implants could be tailored in an on‐demand manner to address specific needs.^[^ ^44^, ^45^, ^46^, ^47^, ^48^ ^]^ Based on the clinical and technological merits above, it is of significant interest to develop functionalized orthopedic implants with osteoimmunomodulatory capabilities for post‐operative osteosarcoma care, which may provide integrative approaches for engineering antitumor immune responses while promoting defect repair.

Based on the considerations above, here we provide a comprehensive review of the principles and designs of osteoimmunomodulatory orthopedic implants for post‐surgery treatment of osteosarcoma. We first analyzed the post‐surgery osteosarcoma immune microenvironment and critically discussed the defects in the osteoimmune system, aiming to highlight risk factors that may potentially foster immune tolerance and retard bone regeneration. Extending from these mechanistic insights, we further summarized the recent advances of osteoimmunomodulatory orthopedic implants in associated areas, focusing on their capability to (1) elicit osteosarcoma‐specific antitumor immune responses to prevent tumor regrowth or recurrence and (2) accelerate new bone formation to facilitate osseointegration or defect healing. A perspective was finally provided on the challenges and potential solutions in this area, with special emphasis on their translation and optimization. We hope that this review may promote the understanding regarding the multifaceted roles of the osteoimmune system in the post‐surgery osteosarcoma defects and provide new strategies for improving osteosarcoma treatment outcomes in the clinic.

## 2 Overview of the Osteoimmune System in the Post‐Surgery Osteosarcoma Microenvironment

According to clinical reports, the osteosarcoma tissues are highly infiltrated with various immune cells, and tumor‐immune interaction would foster a highly complex immune microenvironment that promotes the survival and expansion of osteosarcoma cells.^[^ ^49^, ^50^, ^51^, ^52^ ^]^ In terms of the immunotherapeutic potential, osteosarcoma is generally classified as an immunologically cold tumor, characterized by low infiltration levels of effector T cells as well as significant presence of immunosuppressive cells such as regulatory T cells (Tregs), myeloid‐derived suppressor cells (MDSCs), and tumor‐permissive tumor‐associated macrophages (TAMs).^[^ ^53^, ^54^ ^]^ Specifically, osteosarcoma cells tend to present elevated PD‐L1 expression that markedly promotes the exhaustion of infiltrating CD8+ T cells. Meanwhile, osteosarcoma cells also secrete abundant immunosuppressive factors such as indoleamine 2,3‐dioxygenase (IDO), transforming growth factor β (TGF‐β), and interleukin‐10 (IL‐10), further limiting the proliferation and cytotoxic potential of infiltrating effector T cell populations.^[^ ^55^, ^56^, ^57^, ^58^ ^]^ Alternatively, osteosarcoma cells can program infiltrating macrophages toward tumor‐permissive phenotypes while inhibiting dendritic cell maturation through various intercellular communication pathways such as cytokines, molecular factors, and extracellular vesicles, which may contribute to the T cell ignorance and drive osteosarcoma metastasis. Furthermore, exosomes, cytokines, and chemokines secreted by osteosarcoma cells can recruit immunosuppressive cells such as MDSCs and Tregs to the tumor microenvironment and promote their expansion, which may further reinforce the immunosuppression therein.^[^ ^59^, ^60^, ^61^, ^62^ ^]^ Owing to the immunosuppressive traits, conventional immunotherapeutics such as immune checkpoint inhibitors are mostly ineffective against osteosarcoma in the clinic.

**Figure 1:**
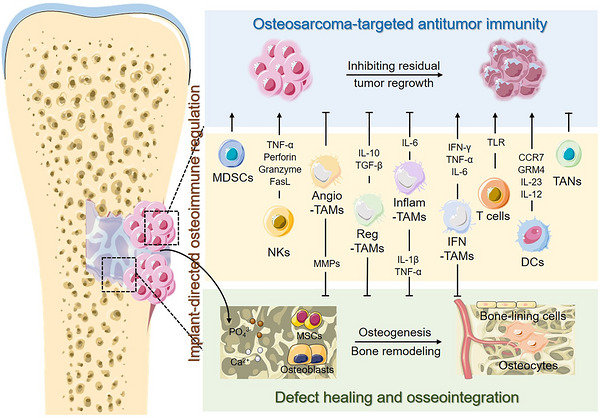
Schematic illustration regarding the therapeutic mechanisms of osteoimmunomodulatory implants for post‐operative osteosarcoma treatment. The functionalized implants can actively interact with residual osteosarcoma cells and the osteoimmune system for coordinating osteosarcoma‐targeted immune responses and defect healing.

On the other hand, the osteoimmune system in osteosarcoma tissues may profoundly affect the bone formation and remodeling events, which is identified as a major contributor to the osteosarcoma symptoms and risks. Notably, the osteosarcoma tissues are mostly under a chronical inflammation state, where the pro‐inflammatory cytokines such as tumor necrosis factor α (TNF‐α), interleukin 6 (IL‐6), and interleukin 1β (IL‐1β) markedly inhibit the osteoblastic differentiation of recruited mesenchymal stem cells (MSCs) while promoting osteoclastogenesis, thus weakening the healthy bone tissues and enhancing the risk of bone fractures.^[^ ^63^, ^64^, ^65^, ^66^ ^]^ It is also notable that the TAMs in osteosarcoma tissues are predominantly skewed toward pro‐tumorigenic and anti‐osteoblastic phenotypes, which secrete TGF‐β and IL‐10 to impair the maturation of residing osteoblasts.^[^ ^67^, ^68^, ^69^, ^70^, ^71^ ^]^ The tumor‐recruited MSDCs present a similar anti‐osteoblastic role by producing excessive ROS and RANK/RANKL‐mediated osteoclastogenesis.^[^ ^72^, ^73^, ^74^ ^]^ These effects would substantially disrupt the bone remodeling programs in osteosarcoma tissues and cause the generation of abundant abnormal bone‐like tissues with aberrant ossification and fibrosis patterns, leading to typical osteosarcoma symptoms including swelling, pain, and functional loss.^[^ ^75^, ^76^, ^77^, ^78^, ^79^, ^80^ ^]^ Furthermore, the osteoimmune system in osteosarcoma tissues could reprogram the recruited MSCs to a pro‐tumorigenic phenotype, showing enhanced secretion capability of PGE2, IDO, and IL‐6 for driving osteosarcoma growth and enhanced propensity to differentiate toward cancer‐associated fibroblasts (CAFs) for promoting metastasis.^[^ ^81^, ^82^, ^83^, ^84^, ^85^ ^]^ It could thus be concluded that the defective osteoimmune system in the osteosarcoma niche would significantly disrupt the bone homeostasis, leading to reduced bone generation and repair capacities (**Figure** **1**).

**Figure 2:**
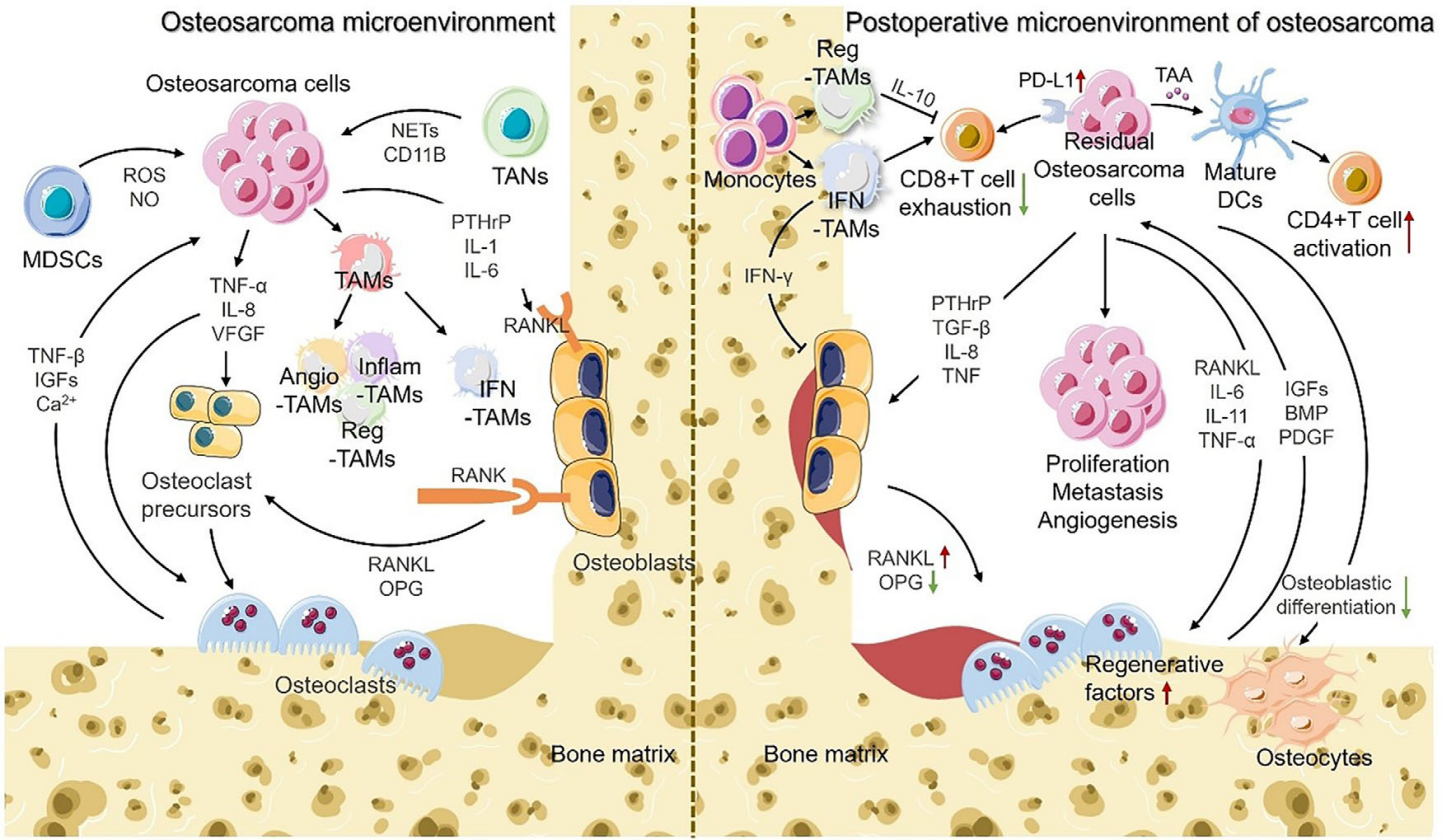
Schematic illustration of the osteosarcoma‐osteoimmune crosstalk for driving tumor progression and bone deterioration (left) and the impact of osteosarcoma surgery on the osteoimmune microenvironment of the defect site (right). The dynamic interaction between residual osteosarcoma cells and wound‐residing immune cells presents critical roles in promoting post‐operative osteosarcoma relapse and bone damage.

Interestingly, the surgical removal of bulk osteosarcoma tissues would induce profound and complex impact on the osteoimmune system in the tumor niche, affecting both the antitumor immune responses and bone metabolism thereof (Figure 2).^[^ ^86^, ^87^, ^88^ ^]^ Of note, the surgery‐induced bone damage and tumor‐associated antigen release would immediately activate acute inflammation responses and trigger the infiltration of abundant innate immune cells including neutrophils and macrophages, leading to a surge in local pro‐inflammatory cytokine levels including TNF‐α, IL‐6 and IL‐1β.^[^ ^89^, ^90^, ^91^, ^92^, ^93^ ^]^ Notably, these pro‐inflammatory cytokines would inhibit the osteogenic function of recruited pro‐regenerative cell populations such as MSCs and osteoblasts while promoting osteoclastogenesis, thus exacerbating the post‐surgery bone deterioration and impeding implant integration.^[^ ^94^, ^95^, ^96^ ^]^ Following the post‐surgery influx of macrophages, they are shaped by various immunostimulatory and immunosuppressive cues including surgery trauma, hypoxia, acidosis and residual osteosarcoma cells, which are eventually skewed toward pro‐angiogenic and immunosuppressive Angio‐TAM, Reg‐TAM and Inflam‐TAM phenotypes,^[^ ^97^ ^]^ thus enhancing the immunosuppressive characteristics of the surgery‐induced bone defects.^[^ ^98^ ^]^ Similarly, enhanced recruitment of Tregs and MSDCs to the post‐surgery defects is often observed in the clinic for the resolution of surgery‐induced acute inflammation, thus adding to the immunosuppression of the bone microenvironment.^[^ ^99^ ^]^ Due to these immunosuppressive mechanisms, although the osteosarcoma surgery may transiently ameliorate the immunosuppressive tumor niche by disrupting the physical and biochemical barriers to facilitate the infiltration of antitumorigenic T cells, the overall antitumor immune responses still remain poor.^[^ ^100^, ^101^, ^102^ ^]^ Meanwhile, retarded post‐operative bone regeneration at the post‐operative defect site is common in the clinic, leading to a series of clinical challenges including prolonged disability and insufficient implant integration.^[^ ^103^, ^104^, ^105^ ^]^ Furthermore, the residual osteosarcoma cells in the wound margin may exploit the post‐surgery immune microenvironment to evade immune surveillance and seed in situ recurrence, which may severely threaten the patient's survival and overall well‐being.^[^ ^106^, ^107^ ^]^

**Figure 3:**
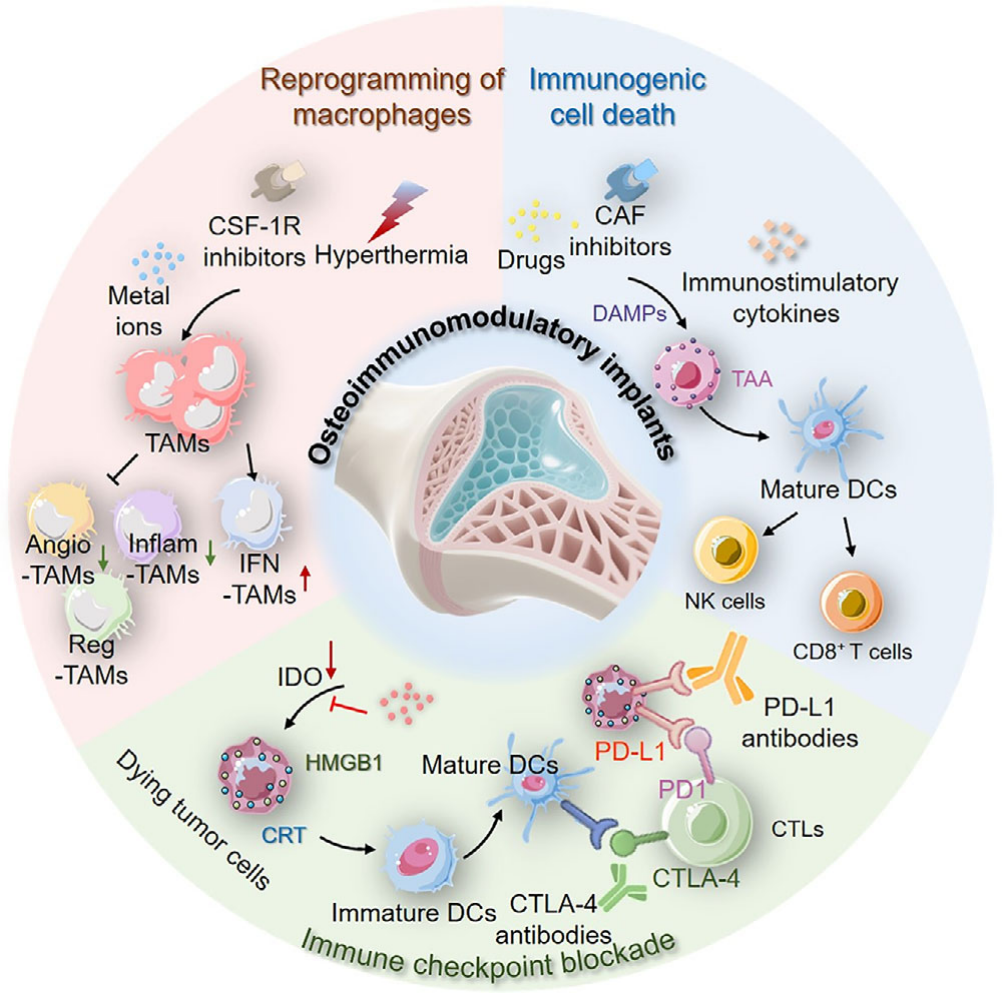
Summary of experimental immunotherapeutic strategies for implant‐directed post‐operative osteosarcoma therapy. The multifunctionality of orthopedic implants allows facile modulation of the bone defect microenvironment and osteosarcoma‐immune cell interaction through (1) TAM reprogramming, (2) ICD induction, and (3) ICB therapy, thus eliciting robust and durable inhibition of osteosarcoma cells in the surgical wound.

Based on the insights above, the osteoimmune system is undoubtedly a double‐edged sword for the post‐operative care of osteosarcoma patients, as it is difficult to simultaneously achieve robust antitumor immune responses and bone regeneration, raising significant challenges for balancing the two therapeutic regimes in a translational setting. On account of the intrinsic capability of osteosarcoma cells to hijack the bone regeneration program to promote tumor repopulation, it is generally necessary to first prioritize on mobilization of antitumor immune responses to ensure adequate elimination of residual osteosarcoma cells before stimulating bone repair, where the restored immune surveillance and established antitumor memory could provide robust protection against osteosarcoma recurrence in the subsequent resolution and healing phases. Consequently, new post‐operative immunoregulatory strategies are urgently needed to restore immunosurveillance and facilitate the follow‐up bone defect healing (**Figure** **3**).

## 3 Immunoregulatory Strategies for Post‐Operative Osteosarcoma Immunotherapy

### 3.1 TAM Reprogramming for Restoring Immune Surveillance

Macrophages are central coordinators in the bone immune microenvironment, presenting major roles in mediating the crosstalk between the local immune responses and osteogenesis.^[^ ^108^, ^109^, ^110^, ^111^ ^]^ Recent high‐throughput analyses reveal a complex and dynamic landscape of osteosarcoma‐infiltrating macrophages, which encompass a spectrum of unique subpopulations with distinct roles in osteosarcoma development and bone metabolism. Based on their molecular signatures and functions, TAMs in osteosarcoma tissues could be generally divided into two generic categories, which are the (1) anti‐tumorigenic TAMs, including interferon‐primed (IFN‐TAMs), as well as (2) pro‐tumorigenic proangiogenic, regulatory, and inflammatory cytokine‐enriched TAMs (Angio‐TAMs, Reg‐TAMs, and Inflam‐TAMs).^[^ ^112^, ^113^, ^114^ ^]^ From a general perspective, recruited monocytes could differentiate into IFN‐TAMs by pro‐immunogenic cues such as IFN‐γ as well as damage‐associated molecular patterns (DAMPs) and dsDNAs released by osteosarcoma cells undergoing immunogenic cell death, of which the hallmarks include elevated expression status of MHC‐II, CD80, CD86, and IRF1. IFN‐TAMs are characterized by an IFN‐driven pro‐inflammatory signature and are typically enriched in osteosarcoma with active T cell infiltration. Notably, IFN‐TAMs are major contributors to antigen presentation and T cell activation in osteosarcoma TME, and their infiltration level shows a marked positive correlation with patient prognosis. Alternatively, osteosarcoma can educate residing macrophages toward tumor‐permissive Angio‐TAM, Reg‐TAM, and Inflam‐TAM phenotypes through a variety of intercellular communication mechanisms.^[^ ^115^ ^]^ For instance, the hypoxic TME and rich VEGF secreted by osteosarcoma cells could readily polarize recruited macrophages toward Angio‐TAMs, which are characterized by high expression levels of VEGFA, PDGFB, ANGPT2, CD206, and SPP1, presenting strong pro‐angiogenesis and vessel remodeling capabilities. Meanwhile, the elevated TME acidity and osteosarcoma cell‐secreted factors such as CSF1, IL‐10, and TGF‐β1 could substantially inhibit the antigen presenting function of macrophages and skew them toward the Reg‐TAM phenotype, which is characterized by elevated IL10, TGFB1, ARG1, CD206, CD163, and IDO1 expression with potent immunosuppressive and T cell exclusion function.^[^ ^116^ ^]^ Furthermore, the chronical inflammation and immunosuppressive features of TME would promote the polarization of macrophages into Inflam‐TAMs, which are enriched with inflammasome activation pathways and show elevated expression of CD163, CD206, and ARG1. Inflam‐TAMs secret abundant TNF‐α and IL‐1β for maintaining the chronical inflammation in osteosarcoma tissues while also potently inhibiting T cell function through PD‐L1 and IDO1 immune checkpoint pathways, thus contributing to osteosarcoma progression and immune evasion.^[^ ^117^, ^118^, ^119^ ^]^ On the other hand, there is concrete evidence that macrophages could strongly affect the whole osteogenesis event through various crosstalk mechanisms.^[^ ^120^, ^121^, ^122^, ^123^ ^]^ Typically, pro‐angiogenesis factors released by Angio‐TAMs can promote the secretion of MMPs to degrade bone matrix in osteosarcoma tissues to exacerbate bone erosion and osteosarcoma invasion. Meanwhile, Reg‐TAMs could secret TGF‐β and IL‐6 to activate RANKL signaling, which promotes osteoclastogenesis in osteosarcoma tissues while suppressing osteoblast differentiation, thus aggravating osteosarcoma‐associated bone loss and deterioration. Inflam‐TAMs show potent pro‐osteolytic capacity through secreting IL‐1β and TNF‐α, both of which are major stimulators of osteoclastogenesis for triggering excessive bone loss. Similarly, IFN‐TAMs may also exert a pro‐osteolytic role through secreting pro‐inflammation cytokines, including IFN‐γ, TNF‐α, and IL‐6. On account of the phenotype‐dependent immunoregulatory and osteoregulatory functions of macrophages in the bone microenvironment, they have demonstrated remarkable promise as therapeutic targets for the post‐operative care of osteosarcoma.

Notably, TAMs in osteosarcoma microenvironment are not terminally differentiated, but rather present significant phenotypical plasticity and can be readily re‐educated into other phenotypes in response to specific biochemical cues. Therefore, it is generally preferable to reprogram tumor‐permissive TAMs into tumor‐suppressive phenotypes rather than killing them for boosting antitumor immunity.^[^ ^124^, ^125^, ^126^ ^]^ Notably, conventional TAM‐reprogramming modalities mostly revolve around key TAM survival and polarization signaling pathways, including TLR, CSF1, and STAT3. For instance, treating tumor‐permissive TAMs with TLR agonists such as CpG could activate TLR7/9 signaling, readily inducing their repolarization toward IFN‐TAM phenotypes to enhance antigen presentation and tumoricidal effects.^[^ ^127^, ^128^, ^129^ ^]^ Similarly, treating tumor‐permissive TAMs with CSF1/CSF1R inhibitors or STAT3 inhibitors could block CSF1/CSF1R axis or STAT3‐signaling network for converting them into tumor‐suppressive phenotypes. Interestingly, recent studies reveal that several bioactive metal ions also present remarkable immunostimulatory effects and play distinct roles in promoting TAM reprogramming. Notably, Mg^2+^ and Zn^2+^ ions mostly act on NF‐κB and TLR signaling pathways to enable anti‐tumorigenic phenotypical transition of TAMs, while Mn^2+^ ions could activate cGAS–STING and NF‐κB signaling in macrophages to convert them into IFN‐TAM phenotypes.^[^ ^130^, ^131^ ^]^ Several studies reveal that iron‐induced ROS stress could also reprogram tumor‐suppressive TAMs into antitumorigenic CD86+TNF‐α+ tumor‐suppressive TAMs, although the actual mechanism remains to be investigated.^[^ ^132^, ^133^ ^]^ In addition, external heating could activate heat shock responses to stimulate macrophage‐intrinsic TLR2/4 signaling as well as stimulating STAT1 phosphorylation to promote IFN‐related gene expression, which could act in a cooperative manner to reprogram TAMs toward IFN‐TAM phenotypes.^[^ ^134^, ^135^ ^]^ Overall, these potential TAM‐reprogramming stimuli provide the biochemical basis for implant‐mediated TAM regulation.

### 3.2 Inducing Immunogenic Cell Death (ICD) of Osteosarcoma Cells for In Situ Vaccination

Despite the tremendous progress in immunotherapy in recent years, the development of effective osteosarcoma immunotherapy has thus far been challenging, which is largely due to the critical lack of consistently targetable surface antigens. From a clinic perspective, osteosarcoma is widely recognized as a highly heterogeneous disease with low intrinsic immunogenicity, which substantially impedes the osteosarcoma recognition and elimination capability of conventional immunotherapeutic modalities.^[^ ^136^, ^137^, ^138^ ^]^ Interestingly, ICD‐based immunostimulatory strategies have attracted significant interest for overcoming these immunoevasive traits.^[^ ^139^, ^140^ ^]^ Typically, ICD can be initiated by specific physical and biochemical stimuli such as ionizing radiation, heat shock, or chemotherapeutics, which will cause the release of tumor‐associated antigens and DAMPs into the tumor microenvironment, thus transforming the immunoevasive tumor cells into in situ vaccines to stimulate tumor‐specific immune responses.^[^ ^141^, ^142^, ^143^ ^]^ For instance, several types of clinically approved anti‐osteosarcoma chemotherapeutics (doxorubicin and cisplatin) can trigger pronounced DNA damage in osteosarcoma cells to initiate a variety of pro‐immunogenic stress responses, including DNA damage responses (DDRs) and endoplasmic reticulum (ER) stress, mitochondrial damage and leakage, ROS elevation, and activation of pro‐inflammatory NF‐κB/MAPK signaling, thus facilitating the release of DAMPs and osteosarcoma‐specific neoantigens to promote T cell priming.^[^ ^144^, ^145^, ^146^ ^]^ Alternatively, physical cues such as heating, ultrasound, and ionizing radiation have also shown ICD inducing capability at varying degrees, thus enabling the spatiotemporally controlled ICD induction using remotely signals.^[^ ^147^, ^148^, ^149^, ^150^, ^151^ ^]^ Typically, heating triggers pro‐immunogenic stress responses, including ROS upregulation, mitochondrial dysfunction, and heat shock responses, where the generated heat shock proteins could act as DAMPs to activate TLR signaling in antigen‐presenting cells. Ultrasound‐mediated cavitation can disrupt biomembrane structures through inducing intense shear stress, which not only promotes ER stress but also facilitates DAMP and antigen leakage. The pro‐immunogenic mechanisms and functions of radiotherapy are similar to DNA‐damaging chemotherapeutics, of which the typical features include ROS elevation, genotoxic responses, ER stress, and mitochondrial dysfunction. Overall, the pro‐immunogenic features of these therapeutic modalities provide promising approaches for enhancing immune surveillance in the post‐operative wound margin.

### 3.3 Immune Checkpoint Blockade for Remodeling Post‐Operative Osteosarcoma Microenvironment

Immune checkpoint blockade therapy (ICB) is a major class of immunotherapy in current oncology practice, which could reinvigorate T cell‐mediated antitumor immune responses through blocking those T cell‐inhibitory signaling pathways such as PD‐L1, cytotoxic T‐lymphocyte‐associated antigen 4 (CTLA‐4), and indoleamine 2,3‐dioxygenase (IDO).^[^ ^152^, ^153^, ^154^ ^]^ From a general perspective, PD‐L1 is frequently upregulated on tumor cell surface, which can bind to PD‐1 on T cell surface to inhibit CD3ζ signaling while activating Akt‐mTOR pathways and phosphatase recruitment, thus impairing TCR signaling and enforcing T cell exhaustion and anergy.^[^ ^155^ ^]^ CTLA4 is commonly expressed on tumor‐infiltrating Tregs and can compete with co‐stimulatory signal CD28 to bind with CD80 and CD86, which would block T cell activation and promote anergy. Furthermore, CTLA4‐binding markedly enhances the expression of pro‐apoptotic Fas and Bim molecules in T cells, further reducing the effector T cell populations in the osteosarcoma microenvironment.^[^ ^156^, ^157^ ^]^ Tumor cells also frequently show upregulated expression of IDO1, which could catalyze the degradation of immunosupportive tryptophan into immunosuppressive kynurenine, thus not only restricting T cell proliferation but also enhancing Treg expansion.^[^ ^158^, ^159^ ^]^ Consequently, a plethora of antibody‐based ICBs have already been used in clinical practice, while several small‐molecule and aptamer‐based ICBs are currently under development.^[^ ^160^, ^161^ ^]^ Although ICBs have significantly improved the therapeutic outcome of multiple cancer indications, including melanoma, non‐small cell lung cancer, Hodgkin lymphoma, etc, osteosarcoma patients generally show unsatisfactory responses to common ICB modalities, ascribing to multiple osteosarcoma‐associated immunosuppressive traits, including low overall T cell infiltrating, limited immunogenicity, excessive presence of immunosuppressor cells, and heterogeneous immune checkpoint expression.^[^ ^162^, ^163^, ^164^ ^]^ Interestingly, the surgery‐induced disruption in osteosarcoma microenvironment could transiently attenuate the immunosuppression by facilitating T cell infiltration, enhancing TAA and DAMP exposure, and recruiting tumor‐suppressive macrophages, which may substantially improve the antitumor immune responses to ICBs and open up new avenues for the post‐operative treatment of osteosarcoma.

The fundamental immunoregulatory strategies analyzed above establish a powerful rationale for the post‐operative management of osteosarcoma, offering potential therapeutic approaches to reshape the hostile wound microenvironment by targeting specific drivers of post‐operative tumor recurrence and osteolysis. Nevertheless, the utility of these agents as systemic agents may cause significant off‐site immunotoxicity, thus warranting the development of localized therapeutic platforms to improve their controllability and specificity.

## 4 Development of Osteoimmunomodulatory Implants for Integrative Post‐Operative Care of Osteosarcoma

Through the convergence of material science and immunology, osteoimmunomodulatory orthopedic implants represent a promising therapeutic platform for the therapeutic modulation of the endogenous osteoimmune system. Indeed, orthopedic implants have long been used in the clinic for treating various forms of bone defects and diseases on account of their mechanical supporting functions. Interestingly, the recent advances in implant technology offer emerging opportunities to further expand their application range in the clinic, which could be achieved through facile integration of bioactive components such as chemotherapeutics, immunoregulatory agents, and regenerative medicine.^[^ ^165^, ^166^, ^167^ ^]^ Currently, the two greatest challenges against osteosarcoma surgery in the clinic are (1) high risk of local relapse due to residual osteosarcoma cells and (2) unhealable bone defects due to surgery trauma. Therefore, an ideal osteoimmunomodulatory implant for post‐operative osteosarcoma treatment should not only possess essential properties and functions for typical orthopedic implants, including biocompatibility, native bone‐mimetic mechanical strength, and osteoinductivity, but also be capable of leveraging the immune microenvironment in the post‐operative wound margin to facilitate the clearance of residual osteosarcoma cells and bone defect repair. In this section, we provide a comprehensive summary of the recent progress in the development of osteoimmunomodulatory orthopedic implants for the integrative engineering of antitumorigenic and pro‐regenerative immune responses in the post‐surgery bone defect, which is outlined according to the specific immunoregulatory mechanisms.

### 4.1 Implant‐Directed Reprogramming of Defect‐Residing Macrophages

**Figure 4:**
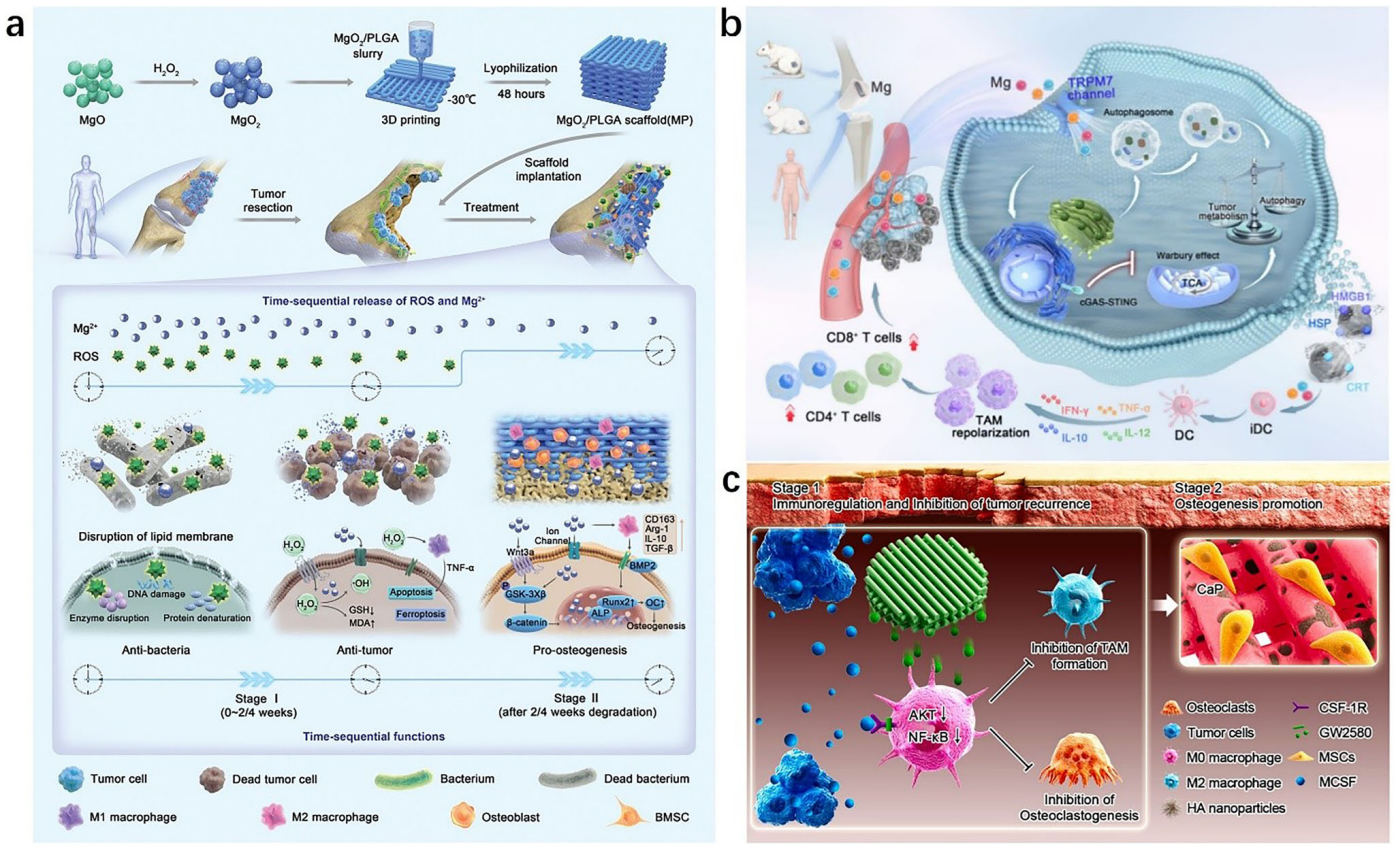
Therapeutic reprogramming of TAMs in post‐operative wound margin for osteosarcoma inhibition and bone regeneration. a) Preparation procedures of the MgO_2_/PLGA composite implant and the time‐sequential macrophage regulatory capabilities for coordinated osteosarcoma inhibition and osteogenesis activities. Reproduced with permission from Ref. [172]. *2023 Wiley‐VCH GmbH*. b) Mechanism of the biodegradable Mg‐based implants for driving autophagy‐dependent osteosarcoma metabolism‐immune crosstalk, leading to efficient macrophage reprogramming for post‐surgery macrophage elimination. Reproduced with permission from Ref. [173, 174]. *2025 Wiley‐VCH GmbH*. c) Mechanism of the 3D printed implants for regulating the post‐operative macrophage microenvironment for cooperative osteosarcoma therapy and defect healing. Reproduced with permission from Ref. [175] *2022 The Authors. Publishing services by Elsevier B.V. on behalf of KeAi Communications Co. Ltd*.

Orthopedic implants have emerged as clinically favorable platforms for the localized reprogramming of macrophages in the post‐surgery bone defects, as they could affect the polarization status and immunological function of macrophages through their intrinsic topological, physical, and chemical properties, as well as mediating in situ drug delivery.^[^ ^168^, ^169^, ^170^, ^171^ ^]^ Specifically, Li et al reported a bioactive and biodegradable MgO_2_/poly (lactide‐co‐glycolide) (PLGA) composite scaffold as a multifunctional bone substitute for post‐surgery osteosarcoma treatment (**Figure** **4**a).^[^ ^172^ ^]^ Of note, MgO_2_ nanoparticles and PLGA precursors were facilely integrated through a unique low‐temperature rapid prototyping (LT‐RP) 3D printing technology, leading to the formation of a hierarchical porous structure with tunable geometries and mechanical properties, allowing a close fit to the complex post‐surgery bone defects in a tailorable manner. Upon installation into the defect site, the MgO_2_ nanoparticles would undergo gradual hydrolysis to release H_2_O_2_ and Mg^2+^ ions into the wound microenvironment in a sustainable manner. In the initial stage of scaffold‐mediated post‐operative osteosarcoma treatment, the release of H_2_O_2_ could inhibit residual osteosarcoma cells through inducing apoptosis and ferroptosis, while Mg^2+^ ions repolarize tumor‐permissive TAMs to a tumor‐suppressive phenotype, thus boosting antitumor immune responses to inhibit osteosarcoma recurrence. Meanwhile, the gradual accumulation of Mg ions in the wound microenvironment would activate the Wnt3a/GSK‐3β/β‐catenin signaling in recruited bone marrow‐derived MSCs, thus promoting their osteoblastic differentiation as well as abolishing macrophage‐mediated excessive inflammation reaction to facilitate bone regeneration. Interestingly, the time‐sequential therapeutic actions of MgO_2_‐derived H_2_O_2_ and Mg^2+^ offers a novel approach to solve the dilemma between the antitumorigenic and osteopromotive function of the defect‐residing macrophages, emphasizing the importance of precisely control the timing of implant‐directed macrophage regulatory effects for maximizing the antitumor and regenerative benefit. A similar strategy was recently reported to enhance the implant‐mediated post‐surgery osteosarcoma therapy by exploiting the immunoregulatory capability of Mg^2+^ ions (Figure 4b).^[^ ^173^, ^174^ ^]^ Here the authors developed a high‐purity biodegradable Mg‐based implant that could not only sustainably release Mg^2+^ ions to induce autophagic cell death of osteosarcoma cells, but also alkalinize the residual tumor tissues to disrupt the anti‐immunogenic tumor metabolic symbiosis, which acted in a cooperative manner to drive tumor‐suppressive macrophage repolarization. Alternatively, Li et al reported a facile in situ macrophage reprogramming strategy for post‐surgery osteosarcoma treatment through implant‐mediated localized delivery of colony‐stimulating factor 1 receptor (CSF‐1R) inhibitor GW2580 (Figure 4c).^[^ ^175^ ^]^ Of note, the authors first prepared a 3D printed calcium phosphate scaffold and coated its surface with a layer of hydroxybutylchitosan/oxidized chondroitin sulfate hydrogel through in situ crosslinking, thus allowing efficient complexation of GW2580 through electrostatic interaction while shielding the pro‐regenerative calcium phosphate contents from the hostile post‐surgery defect microenvironment during early stages of wound healing. The in situ release of GW2580 could inhibit the CSF‐1R and NF‐κB signaling in wound‐residing TAMs and promote their repolarization from tumor‐permissive to tumor‐suppressive phenotypes, simultaneously boosting post‐operative antitumor immune responses while blocking monocyte osteoclastogenesis. In addition, the calcium phosphate components could supply the essential biominerals for supporting bone defect reconstruction during later stages of wound healing. Consequently, the 3D‐printed composite scaffold could simultaneously boost anti‐osteosarcoma immune responses and accelerate bone defect repair. Liu et al developed a multifunctional coating based on Fe‐doped polypyrrole (Fe‐Ppy) and CaO_2_ for the modification of sulfonated polyetheretherketone (SP) implants and used them for the self‐adaptive post‐surgery treatment of osteosarcoma.^[^ ^176^ ^]^ The Fe‐Ppy and CaO_2_ content could synergistically catalyze the production of abundant cytotoxic·OH through (1) Ppy‐mediated regeneration of Fe^2+^ ions through electron pump‐like oxidation and (2) continuous H_2_O_2_ supply through CaO_2_ hydrolysis. Meanwhile, Fe‐Ppy also enabled efficient photothermal therapy of the residual osteosarcoma cells under NIR stimulation. The cooperative chemodynamic‐photothermal therapy could reprogram tumor‐permissive TAMs into tumor‐suppressive phenotypes in the early stages to activate innate and adaptive immune responses. After ameliorating the detrimental tumor microenvironment, the released bioactive Fe and Ca ions could gradually relieve the macrophage‐induced inflammation to boost bone regeneration. Overall, these studies confirmed the potential utility of functional orthopedic implants to support both post‐operative osteosarcoma control and defect healing through regulating phenotypical transition of TAMs.

### 4.2 Implant‐Directed Integrative ICD Induction of Osteosarcoma Cells and Osteogenesis

**Figure 5:**
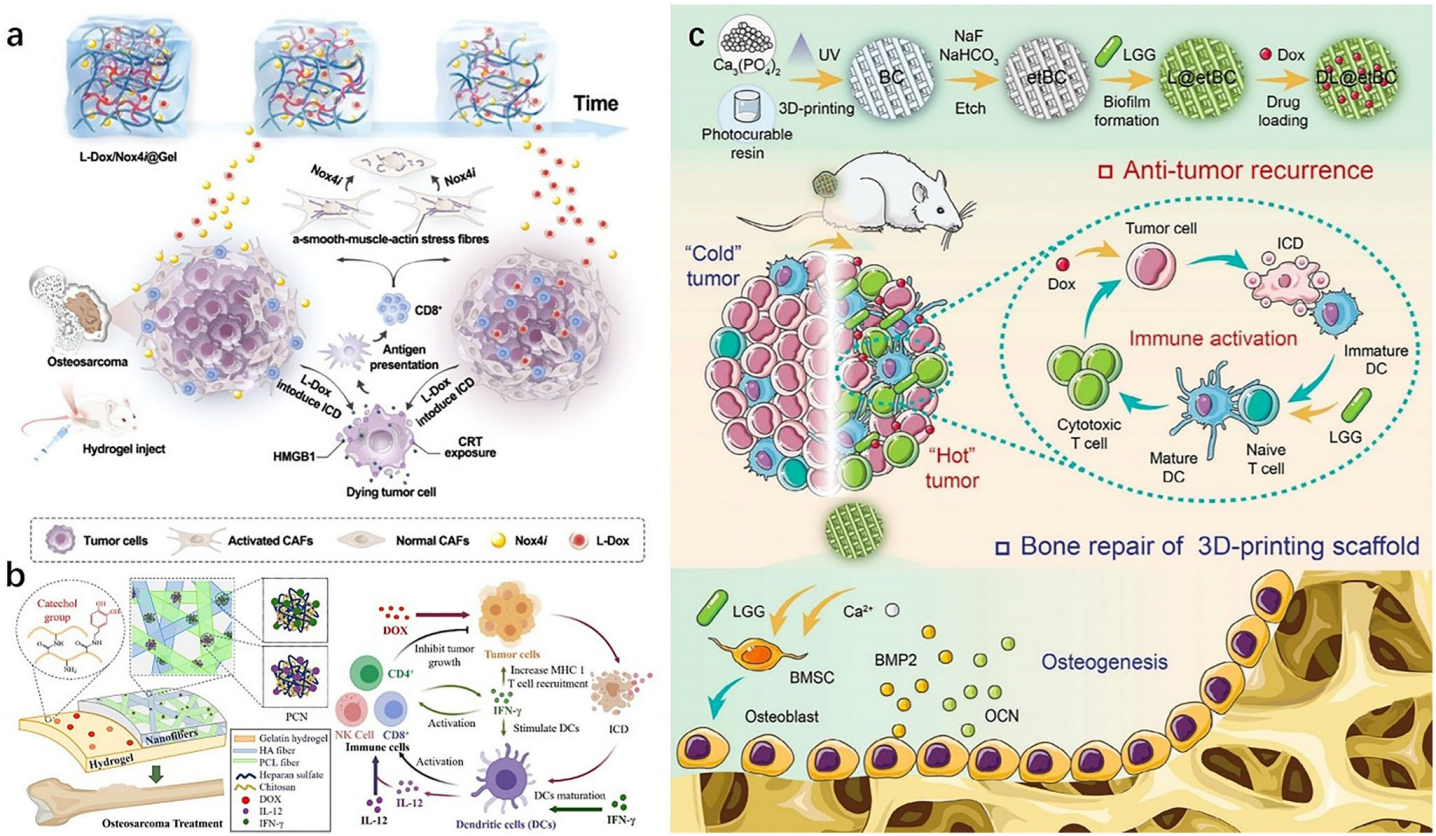
Implant‐directed osteosarcoma‐specific ICD induction using chemotherapeutics for post‐operative osteosarcoma immunotherapy. a) Schematic illustration of the hydrogel implant‐mediated TME remodeling effect through DOX‐mediated ICD of osteosarcoma cells and Nox4i‐mediated CAF‐reprogramming. Reproduced with permission from Ref. [177] *2023 Wiley‐VCH GmbH*. b) Schematic illustration of the double‐layered nanofibrous hydrogel and the DOX‐dependent chemo‐immunotherapy against osteosarcoma in the post‐operative defects. Reproduced with permission from Ref. [178] *2024 Elsevier B.V. All rights reserved*. c) Construction of the LGG‐DOX composite biofilm coating on 3D‐printed bioceramic implants for cooperative anti‐osteosarcoma immunostimulation and bone regeneration. Reproduced with permission from Ref. [179] *2023 Wiley‐VCH GmbH*.

There is increasing interest to modify orthopedic implants for the localized delivery of the ICD‐inducing cues, offering significant advantages for enhancing the precision and efficacy of the elicited immune responses while reducing the risk of potential systemic immunotoxicities. Specifically, Wang et al reported a nanocomposite hydrogel‐based implant to cooperatively induce ICD of osteosarcoma cells and remodel cancer‐associated fibroblast behavior. The hydrogel was fabricated through the crosslinking between carboxymethyl chitosan and 4arm Poly (ethylene glycol) succinimidyl glutarate for the integration of liposomal DOX and the CAF inhibitor Nox4i (**Figure** **5**a).^[^ ^177^ ^]^ The incorporated liposomal DOX and Nox4i components could be gradually released from the hydrogel matrix after implantation into the surgery‐induced bone defects. Nox4i effectively inhibited CAF activity in the defect site, leading to normalization of tumor stromal ECM and reduced secretion of pro‐tumorigenic cytokines for improving T cell infiltration and function. Meanwhile, liposomal DOX could induce ICD of osteosarcoma cells to facilitate TAA and DAMP release. These effects could act in a cooperative manner to evoke robust antitumor immunity for eliminating residual osteosarcoma cells at local and systemic levels. Owing to the treatment‐induced durable osteosarcoma inhibition and the intrinsic function of the implanted hydrogels as supportive matrices for enhancing cell adhesion, proliferation, and osteogenic differentiation, mice in the treatment group showed a much superior bone restoration effect, of which the osteosarcoma‐induced osteolytic bone damage and heterotopic ossification were almost abolished. Lee et al developed a nanofibrous double‐layer hydrogel for the co‐delivery of DOX and immunostimulatory cytokines, including IFN‐γ and IL‐12 (Figure 5b).^[^ ^178^ ^]^ Specifically, the authors loaded DOX into bioadhesive catechol‐modified gelatin hydrogels as the bottom layer, on which an electrospun nanofibrous HA‐PCL coating was generated for integrating IFN‐γ/IL‐12‐loaded heparan sulfate‐chitosan nanoparticles. The double‐layered hydrogel could stably adhere onto the post‐operative bone defects and release DOX and the cytokines. Interestingly, DOX treatment synergized with IFN‐γ/IL‐12 to evoke potent anti‐osteosarcoma immune responses. Owing to its remarkable bone adhesion and osteosarcoma inhibition effects, the double‐layered hydrogel successfully abolished the osteosarcoma‐induced osteolysis and substantially improved bone repair. Alternatively, based on the implant‐adhering and biofilm‐forming properties of *Lactobacillus rhamnosus* GG (LGG), Gu et al developed an LGG‐DOX biofilm‐coated bioceramic scaffold for post‐operative chemo‐immunotherapeutic treatment of osteosarcoma. The LGG biofilms could not only facilitate DOX loading but also synergize with DOX to promote the ICD of residual osteosarcoma cells to elicit systemic antitumor immune responses (Figure 5c). Additionally, the LGG components could reverse senescence of recruited MSCs to promote their proliferation and osteogenesis function, while the spontaneous degradation of the bioceramic scaffold could continuously provide Ca^2+^‐ions to promote the in situ formation and deposition of biominerals. These effects cooperatively enhanced the new bone formation at the defect site for accelerating defect healing.^[^ ^179^ ^]^ Ma et al developed an injectable nanocomposite hydrogel implant for the targeted delivery of cisplatin and MYC oncoprotein inhibitor NHWD‐870, aiming to overcome the intrinsic treatment resistance of MYC‐upregulated osteosarcoma.^[^ ^180^ ^]^ MYC upregulation is a common mutation of osteosarcoma in the clinic, which may markedly enhance the tumor invasiveness and accelerate disease progression while attenuating the response to immunotherapies through impeding T cell infiltration. For this purpose, the authors prepared an injectable hydrogel substrate through the ligation between poly(3‐amino‐4‐methoxybenzoic acid)‐gelatin‐thioketal and 4arm‐PEG succinimidyl glutarate, which were further loaded with NHWD‐870 and IL11Rα‐targeted liposomes encapsulating cisplatin‐loaded mesoporous MnO_2_ nanocores. Owing to the elevated ROS levels in the tumor microenvironment, the thioketal‐containing hydrogel could be gradually degraded to release NHWD‐870 and liposomes into the defects. NHWD‐870‐enabled MYC inhibition could block the downstream CCL2 and IL13 signaling to repolarize TAMs into anti‐tumorigenic phenotypes and enhance T cell infiltration. Meanwhile, the IL11Rα‐targeted liposomes could be selectively taken up by osteosarcoma cells through receptor‐mediated uptake. Here the endogenous GSH would reduce MnO_2_ to Mn^2+^ to catalyze the production of highly toxic·OH through Fenton‐like reaction, which could synergize with concurrently delivered cisplatin to induce pronounced ICD of osteosarcoma cells. Furthermore, the leaked Mn^2+^ ions could promote maturation of tumor‐infiltrating DCs to enhance their antigen‐presenting function. These effects cooperatively boosted the eventual T cell‐mediated antitumor responses for durable inhibition of MYC‐upregulated osteosarcomas in vivo.

**Figure 6:**
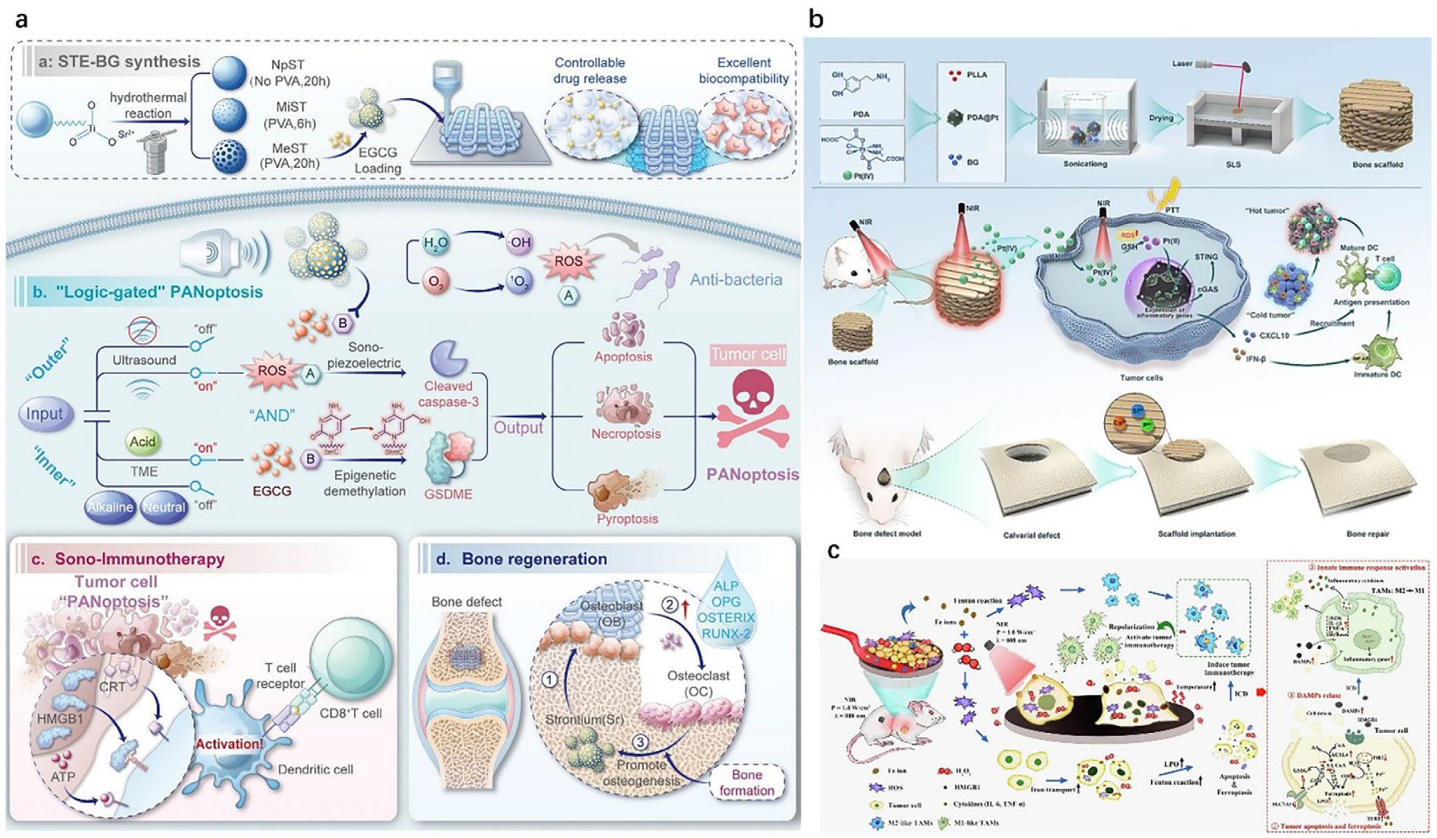
Implant‐dependent physical therapies for ICD induction in osteosarcoma cells in the post‐operative wound margin. a) The sono‐piezoelectric effect of the 3D‐printed BG scaffold for triggering the PANoptosis of osteosarcoma cells, leading to robust antitumor responses in vivo. The degradation products of BG components could further facilitate subsequent defect repair. Reproduced from Ref. [181] *2024 Wiley‐VCH GmbH*. b) Schematic illustration of the photothermal‐induced chemo‐immunotherapy by PLLA/BG/PDA@Pt composite bone scaffolds for post‐operative osteosarcoma care. Reproduced with permission from Ref. [182]. *Copyright 2025, The Author(s)*. c) NIR‐triggerable post‐surgery microenvironment modeling mechanism of the FeOOH/PDA‐Fe‐coated Mg implants for osteosarcoma ferroptosis‐immunotherapy and bone regeneration. Reproduced with permission from Ref. [183]. *2025 Elsevier B.V*. All rights are reserved, including those for text and data mining, AI training, and similar technologies.

For ICD‐induction with physical therapies, Wang et al reported a composite 3D‐printed bioactive glass scaffold integrated with epigallocatechin gallate‐loaded mesoporous piezoelectric SrTiO_3_ nanoparticles for AND‐gated PANoptosis‐dependent post‐operative sono‐immunotherapy of osteosarcoma (**Figure** **6**a).^[^ ^181^ ^]^ On account of the piezoelectric capability of the SrTiO_3_ nanosubstrates, they could produce abundant ROS under external ultrasound input to enhance caspase 3 cleavage, thus triggering PANoptosis of osteosarcoma cells. Meanwhile, the acidic tumor microenvironment could stabilize the released epigallocatechin gallate components and inhibit DNA methyltransferase in osteosarcoma cells to block DNA methylation, which may synergize with the ultrasound‐induced caspase 4 cleavage to enhance osteosarcoma cell fate propensity toward immunogenic pyroptosis, thus facilitating the exposure of TAA and DAMPs to generate T cell‐mediated antitumor immunity. In addition to the potent AND gate antitumor sono‐immunotherapeutic effects, the ultrasound‐triggered piezoelectric ROS production could also eliminate drug‐resistant microbial pathogens in the implantation site to prevent post‐operative implant infection, while the BG component and Sr ions released from the scaffold could markedly promote the proliferation of osteoblasts and stimulate osteogenic genes including ALP, OSTERIX, OPG, RUNX‐2, which massively enhanced the formation of new bone tissues with robust hardness, mineral density and stable integration with original bone tissues. Yan et al conjugated GSH‐reactive Pt(IV) prodrugs onto polydopamine nanoparticles and further integrated them into poly (L‐lactic acid)/bioactive glass matrix via laser sintering (Figure 6b).^[^ ^182^ ^]^ Owing to the photothermal capability of the polydopamine nanoparticles, the Pt(IV) prodrugs could be released upon remote NIR triggers, which were then taken up by residual osteosarcoma cells and activated by endogenous GSH to the bioactive Pt(II) form, thus synergizing with the PDA‐mediated PTT effect to induce ICD of osteosarcoma cells and elicit T cell‐mediated antitumor responses through the cGAS‐STING pathway. Furthermore, owing to the pro‐osteogenic property of BG and PDA components, the composite implants significantly enhanced the osteogenic differentiation of recruited bone marrow‐derived MSCs to facilitate bone defect repair after eliminating residual osteosarcoma cells. Chen et al reported an intelligent dopamine‐Fe^3+^ chelate‐based multifunctional coating on Mg‐based implants, which could not only induce immunogenic ferroptotic death of osteosarcoma cells under NIR‐mediated photothermal effects but also protect the Mg components in the biological environment, leading to enhanced osteosarcoma elimination and long‐term bone defect healing in a coordinated manner (Figure 6c).^[^ ^183^ ^]^ Specifically, the strong ligand‐to‐metal charge transfer between the chelated Fe^3+^ ions and PDA molecules could substantially enhance the photothermal capacity of the compositing coating, which could not only enhance the photothermal heating‐induced cellular damaging effects but also accelerate Fe ion‐catalyzed Fenton reaction to elicit immunogenic ferroptosis, which is conducive to activating the macrophage‐mediated innate antitumor immune responses. Moreover, the Mg‐based implant substrates could be gradually degraded after implantation to release pro‐osteogenic Mg ions into the post‐surgery defects, which could stimulate pro‐osteogenesis pathways, including Wnt/β‐catenin and integrin pathways, thus facilitating bone regeneration without impairing the antitumor immune responses.

The above results collectively supported the therapeutic potential of implant‐induced osteosarcoma ICD for evoking robust and durable anti‐osteosarcoma immunity. Nevertheless, several issues must be considered for implant design and development to ensure their efficacy and safety. Specifically, most of the ICD‐inducing modalities would indiscriminately damage healthy cells in the bone defects and thus release self‐antigens to trigger undesirable autoimmune responses, which may significantly increase the risk of osteodegeneration and other severe symptoms.^[^ ^184^, ^185^ ^]^ Meanwhile, the risk of excessive ICD induction cannot be neglected, as the released DAMPs may disrupt immune homeostasis and provoke overwhelming local and systematic inflammatory side effects.^[^ ^186^, ^187^ ^]^ It is also necessary to avoid prolonged pro‐inflammatory stimulation at the defect site, which may not only cause the premature exhaustion of residing immune cells but also drive inflammatory osteoclastogenesis, leading to impaired antitumor immunity and osteodegenerative efficacy.^[^ ^188^ ^]^ These issues collectively highlighted the importance of optimizing the precision, dosage delivery, and duration of effects for the functionalized orthopedic implants to avoid potential adverse effects.

### 4.3 Implant‐Mediated Immune Checkpoint Blockade for Post‐Operative Osteosarcoma Treatment

**Figure 7:**
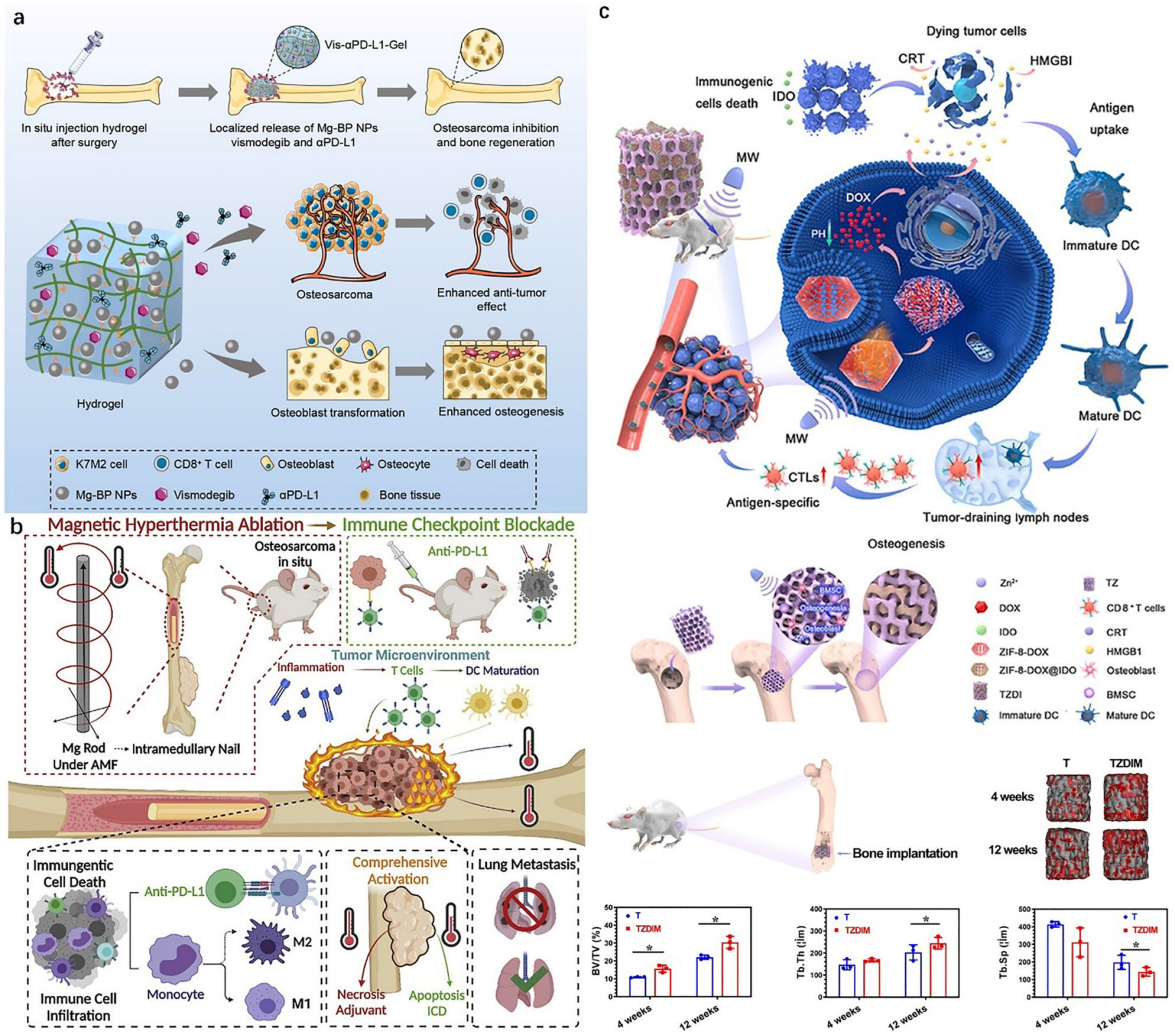
Implant‐dependent ICB for post‐operative osteosarcoma therapy. a) Schematic illustration of the injectable hydrogel‐enabled blockade of PD‐L1 for enhanced osteosarcoma immunotherapy, as well as the Mg‐mediated osteogenic effect. Reproduced with permission from Ref. [189]*. 2024 Elsevier Ltd*. All rights are reserved, including those for text and data mining, AI training, and similar technologies. b) Schematic illustration of the immunostimulatory and pro‐osteogenic mechanism of the Mg‐based rod implants and their synergism with PD‐L1 immune checkpoint inhibition therapy. Reproduced with permission from Ref. [190]. *2023 The Authors. Publishing services by Elsevier B.V. on behalf of KeAi Communications Co. Ltd*. c) Construction and antitumor mechanism of the multifunctional ZIF‐8 coated implants for microwave‐triggered thermal‐immune checkpoint inhibition therapy of osteosarcoma. Reproduced with permission from Ref. [191]. *2023 Published by Elsevier Ltd*.

On account of TME remodeling capacity of ICB therapy, orthopedic implants with ICB functions show significant promise for engineering anti‐osteosarcoma immune responses in the post‐operative defects. Typically, Chu et al developed an injectable self‐healing hydrogel through the supramolecular interaction between bisphosphonate‐modified hyaluronic acid and self‐assembled Mg‐bisphosphonate nanoparticles, which allowed facile integration of PD‐L1 antibodies (aPD‐L1) and a hedgehog pathway antagonist vismodegib while also enabling close fit to the irregular post‐surgery bone defects (**Figure** **7**a).^[^ ^189^ ^]^ The hydrogel‐mediated delivery of aPD‐L1 and vismodegib led to cooperative inhibition of tumor‐intrinsic PD‐L1 and hedgehog signaling pathways, significantly promoting the activation of anti‐tumorigenic CD8+ T cells for inhibiting primary osteosarcomas while building up systemic anti‐osteosarcoma immune memory. On the other hand, the release of Mg^2+^ ions could promote the osteoblastic differentiation of recruited MSCs through stimulating Wnt/β‐catenin and promoting BMP‐2 secretion, thus facilitating the bone reconstruction at the defect site. Ge et al reported a biodegradable Mg‐based implant with dual eddy thermal effect with combined ICB treatment for post‐operative osteosarcoma inhibition and bone regeneration (Figure 7b).^[^ ^190^ ^]^ Specifically, the Mg rod were inserted into the bone defects and agitated using an external alternating magnetic field to generate a localized magnetic hyperthermia field through eddy thermal effect, which could induce osteosarcoma ICD and enhance the tumor infiltration of T cells and repolarization of TAMs into antitumorigenic phenotypes, thus synergizing with the administered aPD‐L1 to evoke potent osteosarcoma‐specific immunity. Similar to the other reports employing Mg‐based biomaterials, the Mg rod could also release pro‐regenerative Mg ions to improve defect healing. Alternative to the usage of PD‐L1 inhibitors, Ma et al employed microwave‐responsive zeolitic imidazolate framework 8 (ZIF‐8) nanoscale metal–organic framework (MOF) for the loading of doxorubicin and an IDO‐specific inhibitor, which was then integrated into 3D‐printed Ti implants for post‐operative osteosarcoma treatment (Figure 7c).^[^ ^191^ ^]^ Notably, the ZIF‐8 nanoMOF could act as a transducers and generate heat under remote microwave stimulation, which could not only directly damage osteosarcoma cells but also stimulate the locoregional release of DOX and IDO inhibitor. Here the DOX and microwave heating effect could synergistically induce the ICD of osteosarcoma cells for the activation of cytotoxic CD8+ T cells, while the concurrently released IDO inhibitor could further block the pro‐tumorigenic IDO signaling to alleviate the T cell inhibition effect, leading to significant enhancement in the robustness and durability of the evoked adaptive anti‐osteosarcoma immune responses. Furthermore, the microwave heating would also induce the gradual degradation of the ZIF‐8 nanoMOFs to release Zn^2+^ ions, which stimulated the osteoblastic differentiation of MSCs by stimulating osteogenic genes, including alkaline phosphatase (ALP), Runx2, and osteocalcin, thus promoting new bone formation at the bone‐implant interface to facilitate osseointegration of the Ti implants. Still, while the above studies collectively supported the immunotherapeutic potential of implant‐assisted ICB for post‐operative osteosarcoma treatment, the ICB‐induced immune responses may potentially promote inflammatory osteoclastogenesis, thus impairing the defect reconstruction and implant integration efficacy. Current studies in this area reveal two main strategies to ameliorate the potential anti‐regenerative effect of ICB osteosarcoma therapy, including (1) optimizing the timing of ICB intervention to reduce the anti‐healing impact and (2) integration of additional pro‐osteogenic components or functions to counteract the ICB‐induced bone resorption, which have demonstrated preliminary success in balancing the implant‐mediated antitumor and defect healing performance.

In summary, the immunomodulatory capabilities of engineered orthopedic implants provide a viable solution to address the current dilemma between antitumor immunity and bone defect healing in post‐operative osteosarcoma management, showing promise to balance the activation and resolution of immune responses in the post‐operative bone microenvironment.^[^ ^192^, ^193^, ^194^, ^195^, ^196^, ^197^ ^]^ Indeed, rationally engineered implants allow facile control over the osteoimmune crosstalk through interacting with infiltrating immune cells, osteoblasts/osteoclasts, MSCs, and residual osteosarcoma cells, thus enabling effective engineering of the immune responses in the post‐operative defects through controlling their spatiotemporal traits. While these technologies are still in their infancy, they are well‐suited to address the complex heterogeneity of post‐operative wound margins, presenting new approaches for promoting long‐term patient survival and functional recovery with clinical promise.

## 5 Perspectives and Challenges of Implant‐Dependent Post‐Operative Osteosarcoma Therapy

Despite their significant potential for post‐operative osteosarcoma treatment, current research in this area also identifies several major challenges that may impair the translation of otherwise promising osteoimmunomodulatory implants under clinically relevant conditions,^[^ ^198^, ^199^, ^200^ ^]^ which are closely associated with their development and application, including the complexity of osteoimmunoregulation, drug target selection, manufacturing, and pre‐clinical investigation. These challenges are discussed in this section with potential solution strategies.

### 5.1 Resolving the Conflict between Antitumor and Pro‐Regenerative Immunity through Dynamic Regulation of Implant‐Mediated Localized Drug Delivery

The divergent impacts of the osteoimmune system on tumor inhibition and osteogenesis in the post‐surgery bone microenvironment are considered central challenges against the development of effective osteoimmunoregulatory implants. Indeed, accumulating studies collectively suggest that antitumor immunity and pro‐osteogenic immune responses are immunologically incompatible.^[^ ^103^, ^201^, ^202^ ^]^ From a general perspective, effective anti‐osteosarcoma immunotherapy relies on the successful activation of type‐1 immune responses, of which the hallmarks include enhanced tumor‐suppressive macrophage polarization, elevated production of pro‐inflammatory cytokines, and abundant infiltration of NK cells, Th1 cells, and cytotoxic CD8+ T cells. In contrast, osteogenesis is generally associated with anti‐inflammatory immune responses, relying on the coordination of pro‐regenerative macrophages, Tregs, and MDSCs to orchestrate a regenerative immune milieu. Moreover, the post‐operative wound of osteosarcoma is not a uniform and static tissue but rather a complex and dynamic ecosystem with marked temporal and spatial heterogeneity. On one hand, the post‐operative wound microenvironment is constantly evolving without therapeutic intervention, which would first undergo an initial inflammation phase and then enter an immunosuppressive state following the re‐education of recruited immune cells by residual osteosarcoma cells, eventually leading to tumor regeneration and bone remodeling. On the other hand, the post‐operative wound margin is also not uniform and contains a mosaic of microenvironmental niches with varying degrees of vascularization, immune cell infiltration, residual osteosarcoma cells, and bone regenerating cells, posing significant obstacles for achieving efficient post‐operative osteosarcoma inhibition and wound regeneration. Interestingly, the recent advances in stimuli‐responsive implants offer new avenues to overcome heterogeneity‐associated challenges by providing localized, on‐demand, and adaptable therapeutic interventions. Typically, the stage‐wise transition of the post‐operative wound margin is usually accompanied by significant alterations in local biochemical cues, which frequently present enhanced acidity and ROS levels in the initial inflammation phases as well as elevated expression levels of certain MMPs in the resolution phase. Consequently, aligning drug release profiles with the post‐operative wound healing sequence could facilitate the on‐time release of immunotherapeutics, which is crucial for adequate elimination of residual osteosarcoma cells in the inflammation phages and accelerating wound healing in the resolution phase. For instance, as residual osteosarcoma cells often reside in acidic or immunosuppressive niches, it is preferable to develop therapeutic implants that could respond specifically to these biochemical cues to release the immunotherapeutics, thus enhancing the pin‐point osteosarcoma elimination efficacy while reducing off‐target immunotoxicity.^[^ ^203^, ^204^, ^205^ ^]^ Of note, endowing functional implant systems with specific responsiveness to external triggers, including light, ultrasound, and magnetic field, may further add to their programmability, which may substantially improve the temporal‐spatial precision of the drug delivery process.^[^ ^206^, ^207^ ^]^ Overall, stimuli‐responsive implants represent a rational and balanced approach to match the therapeutic activities with the temporally and spatially heterogeneous post‐operative wound margin of osteosarcoma, providing a viable approach for solving the conflict between antitumor and pro‐regenerative immunity. Nevertheless, further research effort is still required to improve its potency and safety under clinically relevant conditions.

### 5.2 Rational Selection of Drug Targets for Overcoming Osteosarcoma Heterogeneity

Osteosarcoma is a highly heterogeneous disease with varying immune microenvironmental traits. While the surgery has removed bulk osteosarcoma tissues to alleviate the macroscopic heterogeneity, the post‐operative wound still presents high complexity at the molecular and cellular levels, which strongly affects immune surveillance and in situ bone regeneration. Indeed, osteosarcoma cells are well known for their heterogeneous genetic makeup due to intrinsic genomic instability, making it extremely difficult to screen shared drug targets. This issue is further complicated by the dynamic alterations in the immune composition and signaling network after osteosarcoma surgery, which are infiltrated by residual tumor‐permissive TAMs and recruited monocytes, as well as a cocktail of biochemical cues with potentially contradictory functions. These traits markedly challenge the development of a generally applicable implant design for post‐operative osteosarcoma care, necessitating a more personalized and adaptable approach to adequately accommodate patient‐specific and microenvironmental differences. Specifically, it is important to first clarify the immune composition, histological types, and osteosarcoma genetic makeup through multi‐omics analysis on biopsy and resection samples, which is crucial for revealing tumor epitope immunogenicity as well as potential therapeutic vulnerabilities. For instance, osteosarcoma cells frequently present elevated MYC oncogene expression to block T cell responses,^[^ ^208^ ^]^ to overcome its negative impact on post‐operative osteosarcoma immunotherapy, it would be preferable to combine the selected immunotherapeutics with MYC blockade for patients with high MYC levels.^[^ ^209^ ^]^ Alternatively, post‐operative wounds with excessive hypoxia would stimulate angiogenesis to promote aberrant new vessel formation, which is detrimental for T cell infiltration. Therefore, incorporating anti‐angiogenic agents into therapeutic implants could amplify the antitumor efficacy of co‐delivered immunotherapeutic modalities.^[^ ^210^ ^]^ On the other hand, in view of the heterogeneous expression of osteosarcoma‐associated surface antigens across different subclones, it is usually preferable to expose the full spectrum of osteosarcoma‐associated neoantigens through inducing ICD using selective chemotherapeutics, photothermal therapy, or radiotherapy in an in situ vaccination approach instead of relying on pre‐determined antigens. Furthermore, instead of monoblock implant designs, it would be beneficial to adapt a modular strategy for implant manufacturing where the major therapeutic components are interchangeable, allowing facile customization and adaptation to address patient‐specific needs.^[^ ^209^ ^]^ Overall, it is anticipated that the combination of multi‐omics technology, novel immunotherapeutics/regenerative medicine, and advanced implant engineering offers significant promise for improving osteosarcoma treatment in the clinic.

### 5.3 Implant Types and Manufacturing

**Figure 8:**
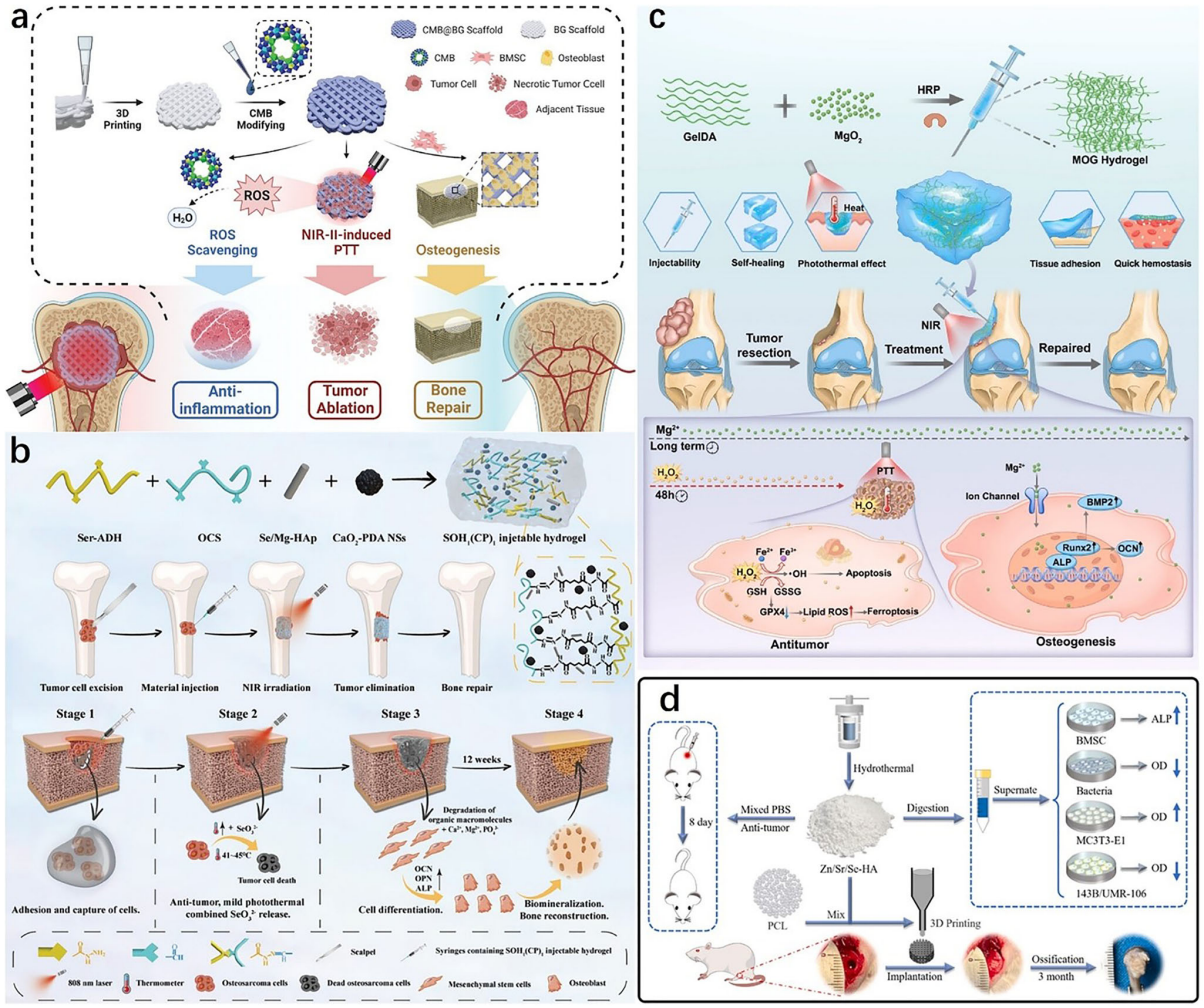
Wound‐conforming implant technologies for improving post‐operative osteosarcoma care. a) Construction of 3D‐printable bioactive glass scaffold for the integration of photothermal catalytic wheel‐like nanocrystals for post‐operative NIR‐II‐triggerable osteosarcoma ablation therapy and defect reconstruction. Reproduced with permission from Ref. [218]. *2024 Wiley‐VCH GmbH*. b) Schematic illustration of the preparation of the injectable hydrogel implants with photothermal and ion release capabilities for osteosarcoma‐related bone defect repair. Reproduced with permission from Ref. [219]. 2024 Wiley‐VCH GmbH. c) Preparation of the MgO_2_‐integrated injectable hydrogel for post‐operative chemodynamic‐photothermal osteosarcoma therapy and bone repair. Reproduced with permission from Ref. [220]. 2024 Elsevier Ltd. All rights are reserved, including those for text and data mining, AI training, and similar technologies. d) Preparation process of the metal ion‐doped hydroxyapatite‐based implants through 3D printing for post‐operative selenide‐based osteosarcoma therapy, wound disinfection, and defect healing. Reproduced with permission from Ref. [221]. *2023 The Authors. Publishing services by Elsevier B.V. on behalf of KeAi* *Communications Co. Ltd*.

Manufacturing and quality control of osteoimmunomodulatory orthopedic implants remain major concerns for their broad application in the clinical context. Notably, as the post‐operative wound margin contains both healthy osteocytes (predominant population) and osteosarcoma cells (minor presence), uncontrolled drug delivery to the wound margin would result in negligible drug deposition in osteosarcoma cells, leading to significantly attenuated osteosarcoma inhibition efficacy and severe side effects. A potential strategy to enhance the cellular specificity of the implant‐mediated localized drug delivery is to modify the therapeutic contents with cell‐targeting ligands in an antibody‐drug‐conjugate‐like manner, which may improve their targeting specificity against selected cell populations. However, additional engineering of drug molecules may induce unpredictable changes in their pharmacological activities while significantly reducing the overall cost‐effectively. Instead, the therapeutic contents could be first loaded into engineered nanocarriers before integration into the implant substrate, which would be preferably internalized by targeted cell populations to reduce non‐specific uptake without invasive modification of the drug molecules.^[^ ^211^, ^212^, ^213^ ^]^ Another practical challenge in implant manufacturing is that mainstream orthopedic implants exist mostly in predetermined sizes and shapes, while the anatomic features of post‐surgery bone defects may vary significantly among different patients, which would not only require complex adjustment of the implants but also impair their therapeutic consistency and efficacy, leading to insufficient osteosarcoma inhibition or bone regeneration. Consequently, it is often necessary to design and manufacture the implants on a case‐by‐case basis to ensure a close fit to the post‐operative defects, aiming to improve implant stability and immunomodulatory efficacy. The recent advances in material technology offer novel opportunity for the development of shape‐conforming orthopedic implants with tunable interfacial properties and therapeutic functionality, of which the leading examples include 3D printing technology and injectable hydrogels. These emerging technologies enable the facile manufacturing of patient‐specific implant designs with complex geometries and predictable therapeutic activities (**Figure** **8**).^[^ ^214^, ^215^, ^216^, ^217^, ^218^, ^219^, ^220^, ^221^ ^]^ Notably, injectable hydrogels are a class of emerging biomaterials with excellent functional versatility.^[^ ^222^, ^223^ ^]^ Compared with conventional bulk hydrogels based on permanent covalent bonding, injectable hydrogels are generally prepared through the crosslinking of polymeric components using reversible dynamic bonds or supramolecular interactions, which could thus exist as solutions before injection but undergoes rapid crosslinking in biological environment to form 3D hydrogel networks, thus enabling minimally invasive delivery to deep post‐operative bone cavities while also potentiating close fitting to the irregular wounds. Interestingly, the large pore space and versatile polymer chemistry allow efficient accommodation of immunoregulatory and regenerative agents via simple procedures. Nevertheless, injectable hydrogels mostly lack mechanical strength due to the weak bonding strength and are thus not suitable for treating large or high‐force‐loading defects. On the other hand, 3D printing is a computer‐controlled additive manufacturing technology, which allows the precise fabrication of bioactive implants with tailorable external shapes and internal architectures based on the CT or MRI scanning of the post‐operative cavity.^[^ ^224^, ^225^, ^226^ ^]^ 3D printed scaffold can be fabricated using a variety of precursor materials, including polymers, metals, ceramics, and organic‐inorganic hybrid materials, which are not only capable of localized delivery of therapeutic agents but also offer high mechanical strength for supporting damaged loading‐bearing bones.

### 5.4 Discrepancies between Common Animal Models and Human Osteosarcoma Pathology

**Figure 9:**
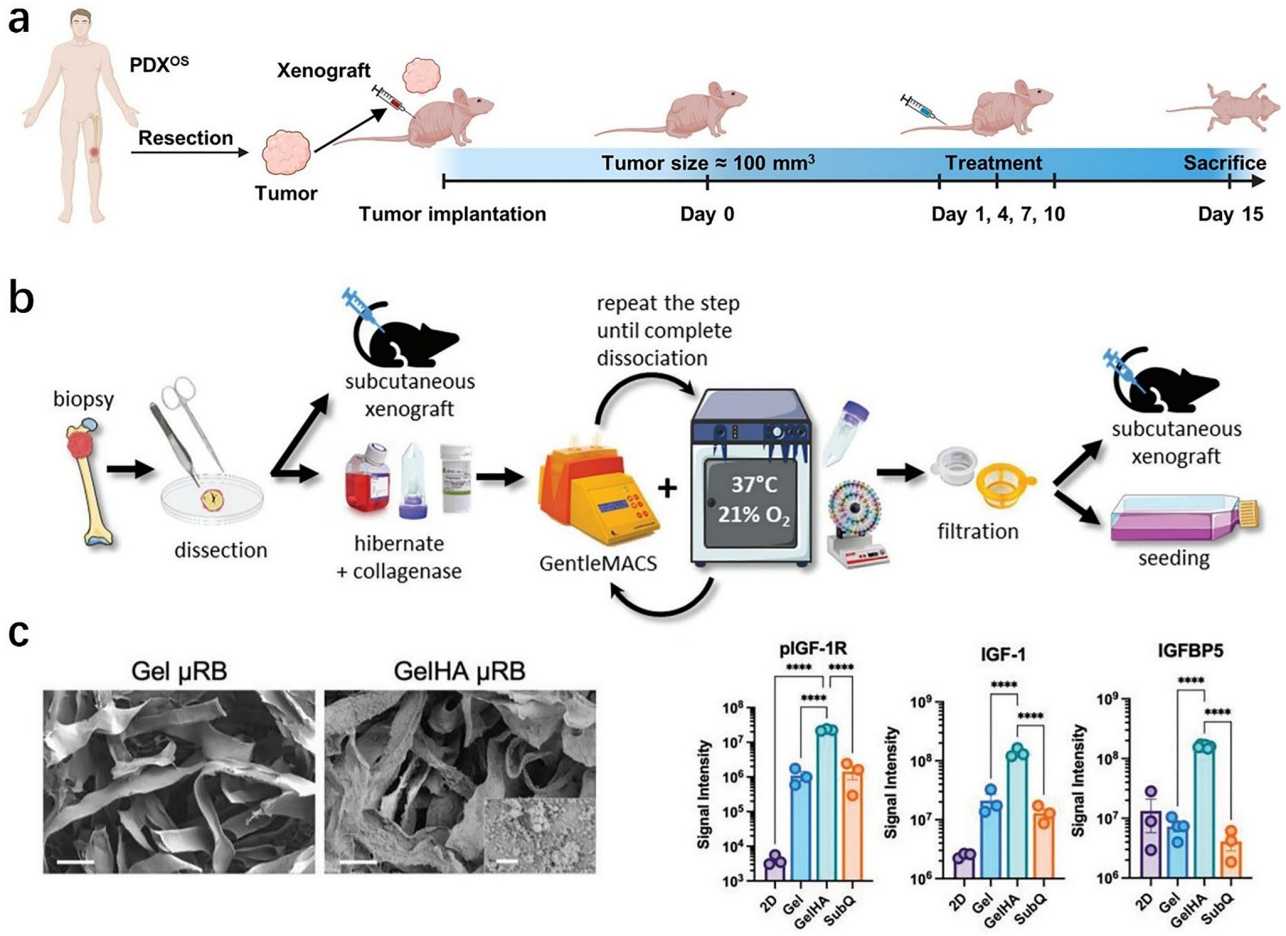
Emerging technologies for constructing osteosarcoma surgery models with enhanced clinical representativeness. a) Construction scheme of the patient‐derived osteosarcoma model on mice. Reproduced with permission from Ref. [227]. *Copyright 2024, The Author(s)*. b) Scheme for the establishment of the 3D osteosarcoma model for recreating the high‐grade osteosarcoma immune microenvironment and ECM structures. Reproduced with permission from Ref. [228]. 2022 Wiley‐VCH GmbH. c) Bone‐mimetic structure of the gelatin‐hydroxyapatite microribbon scaffolds and their osteoimmune regulatory capabilities. Reproduced with permission from Ref. [229]. *2022 Wiley‐VCH GmbH*.

Current osteosarcoma research mostly employs syngeneic or xenograft models on small animals such as rats or mice, which tend to be more immunogenic than the immunoevasive and immunotolerant osteosarcomas in real‐life patients while lacking phenotypical and immunological heterogeneity. Meanwhile, current post‐surgery osteosarcoma models often fail to recapitulate major clinical traits of the post‐surgery osteosarcoma microenvironment, including surgery‐induced osteoimmune remodeling, residual micrometastases, and impaired bone regeneration capacity. The discrepancy between preclinical anti‐osteosarcoma research and real‐life conditions highlights the urgent need for the establishment of more advanced ex vivo and orthotopic animal models to improve their clinical relevance and translatability. For instance, Zhang et al developed a patient‐derived osteosarcoma xenograft mouse model through implanting human osteosarcoma tissue fragments into immunodeficient mice to recapitulate the hypoxic and heterogeneity traits of human osteosarcoma for evaluating the therapeutic activity of their GSH oxidation nanoreactor system (**Figure** **9**a).^[^ ^227^ ^]^ Pierrevelcin et al developed a high‐grade osteosarcoma‐3D model through engineering patient‐derived osteosarcoma cells in collagen‐chitosan scaffolds, which could not only replicate the bone microstructures but also mimic major clinical traits of osteosarcoma, including hypoxia, TAM‐osteosarcoma interaction, and tumor heterogeneity (Figure 9b).^[^ ^228^ ^]^ Díaz et al developed a 3D osteosarcoma model through integrating patient‐derived osteosarcoma cells into gelatin‐hydroxyapatite microribbon scaffolds, which could replicate the bone‐mimetic cues for regulating osteosarcoma signaling and therapy responses (Figure 9c).^[^ ^229^ ^]^ These studies may provide emerging opportunities for elucidating the complex immune cell/osteosarcoma cell/bone matrix interaction with enhanced clinical relevance.

## 6 Conclusion

The past few decades have witnessed a remarkable surge in osteoimmunological research, which has significantly reshaped the treatment paradigms for a broad spectrum of bone diseases. Particularly for the therapeutic intervention against osteosarcomas, the growing understanding of the dynamic interaction among the native bone, osteosarcoma tissue, and the immune system underscores the crucial role of the osteoimmune system in leveraging anti‐osteosarcoma immune responses and osteogenesis, providing a strong mechanistic basis for the development of multifunctional orthopedic implants to improve osteosarcoma treatment outcomes in a clinic context. In this review, we critically analyzed the osteosarcoma immune microenvironment as well as the impact of surgical removal of bulk tumors on the osteoimmune system therein, followed by a comprehensive account of the recent progress in the field of osteoimmunomodulatory orthopedic implants for the on‐demand engineering of anti‐tumorigenic and pro‐osteodegenerative immune responses in the post‐surgery wound microenvironment. This integrative approach holds substantial promise to overcome the limitations of conventional osteosarcoma therapy for improving patient survival and defect regeneration.

## Conflict of Interest

The authors declare no conflict of interest.

## References

[1] H. C. Beird , S. S. Bielack , A. M. Flanagan , J. Gill , D. Heymann , K. A. Janeway , J. A Livingston , R. D. Roberts , S. J. Strauss , R. Gorlick , Nat. Rev. Dis. Primers 2022, 8, 77. 36481668 10.1038/s41572-022-00409-y

[2] S. Cole , D. M. Gianferante , B. Zhu , L. Mirabello , Cancer 2022, 128, 2107. 35226758 10.1002/cncr.34163 PMC11647566

[3] R. L. Siegel , A. N. Giaquinto , A. Jemal , Cancer J. Clin. 2024, 74, 12. 10.3322/caac.21820 38230766

[4] F. Bray , M. Laversanne , H. Sung , J. Ferlay , R. L. Siegel , I. Soerjomataram , A. Jemal , CA: Cancer J. Clin. 2024, 74, 229. 38572751 10.3322/caac.21834

[5] B. Han , R. Zheng , H. Zeng , S. Wang , K. Sun , R. Chen , L. Li , W. Wei , J. He , J. Natl. Cancer Cent. 2024, 4, 47. 39036382 10.1016/j.jncc.2024.01.006 PMC11256708

[6] P. S. Meltzer , L. J. Helman , N. Engl. J. Med. 2021, 385, 2066. 34818481 10.1056/NEJMra2103423

[7] S. J. Strauss , A. M. Frezza , N. Abecassis , J. Bajpai , S. Bauer , R. Biagini , S. Bielack , J. Y. Blay , S. Bolle , S. Bonvalot , I. Boukovinas , J. V. M. G. Bovee , K. Boye , B. Brennan , T. Brodowicz , A. Buonadonna , E. De Álava , A. P. Dei Tos , X. Garcia Del Muro , A. Dufresne , M. Eriksson , F. Fagioli , A. Fedenko , V. Ferraresi , A. Ferrari , N. Gaspar , S. Gasperoni , H. Gelderblom , F. Gouin , G. Grignani , Ann. Oncol. 2021, 32, 1520. 34500044 10.1016/j.annonc.2021.08.1995

[8] R. Belayneh , M. S. Fourman , S. Bhogal , K. R. Weiss , Curr. Oncol. Rep. 2021, 23, 71. 33880674 10.1007/s11912-021-01053-7

[9] T. Shim , Y. Chillakuru , C. Darwish , E. Chalif , D. Strum , D. A. Benito , C. F. Mulcahy , A. Monfared , Head Neck 2021, 43, 3294. 34272901 10.1002/hed.26817

[10] R. van Ewijk , N. Herold , F. Baecklund , D. Baumhoer , K. Boye , N. Gaspare , S. B. Harrabif , L. M. Havemana , S. Hecker‐Noltingg , L. Hiemcke‐Jiwah , V. Martini , C. M. Fernándezj , E. Palmerinik , M. A. J. van de Sandea , S. J. Straussm , S. S. Bielackg , L. Kager , Oncol 2023, 2, 100029.

[11] J. S. Biermann , A. Hirbe , S. Ahlawat , N. M. Bernthal , O. Binitie , S. Boles , B. Brigman , A. K. Callan , C. Cipriano , L. D. Cranmer , J. Davis , E. Donnelly , M. Ferguson , A. Graham , J. Groundland , M. Hess , S. M. Hiniker , M. L. Hoover‐Regan , J. L. Hornick , B. Jonard , J. B. Kuechle , D. Lindskog , J. L. Mayerson , S. V. McGarry , C. D. Morris , D. Olson , P. S. Rose , V. M. Santana , R. L. Satcher , H. Schwartz , J. Natl. Compr. Cancer Netw. 2025, 23, 250017. 10.6004/jnccn.2025.0017 40203873

[12] A. K. LeBlanc , C. N. Mazcko , A. Cherukuri , E. P. Berger , W. C. Kisseberth , M. E. Brown , S. E. Lana , K. Weishaar , B. K. Flesner , J. N. Bryan , D. M. Vail , J. H. Burton , J. L. Willcox , A. J. Mutsaers , J. P. Woods , N. C. Northrup , C. Saba , K. M. Curran , H. Leeper , H. Wilson‐Robles , B. G. Wustefeld‐Janssens , S. Lindley , A. N. Smith , N. Dervisis , S. Klahn , M. L. Higginbotham , R. M. Wouda , E. Krick , J. A. Mahoney , C. A. London , Clin. Cancer Res. 2021, 27, 3005. 33753454 10.1158/1078-0432.CCR-21-0315 PMC8172450

[13] N. Gaspar , R. Venkatramani , S. Hecker‐Nolting , S. G. Melcon , F. Locatelli , F. Bautista , A. Longhi , C. Lervat , N. Entz‐Werle , M. Casanova , I. Aerts , S. J. Strauss , E. Thebaud , B. Morland , A. C. Nieto , P. Marec‐Berard , M. Gambart , C. Rossig , C. E. Okpara , C. He , L. Dutta , Q. Campbell‐Hewson , Lancet Oncol. 2021, 22, 1312. 34416158 10.1016/S1470-2045(21)00387-9

[14] Y. Jiang , J. Wang , M. Sun , D. Zuo , H. Wang , J. Shen , W. Jiang , H. Mu , X. Ma , F. Yin , J. Lin , C. Wang , S. Yu , L. Jiang , G. Lv , F. Liu , L. Xue , K. Tian , G. Wang , Z. Zhou , Y. Lv , Z. Wang , T. Zhang , J. Xu , L. Yang , K. Zhao , W. Sun , Y. Tang , Z. Cai , S. Wang , Nat. Commun. 2022, 13, 7207. 36418292 10.1038/s41467-022-34689-5 PMC9684515

[15] W. Zhang , C. Yin , L. Qi , Z. Liu , R. Xu , C. Tu , Z. Li , Adv. Sci. 2025, 12, 2410937. 10.1002/advs.202410937 PMC12021087 40019400

[16] G. Bacci , F. Bertoni , A. Longhi , S. Ferrari , C. Forni , R. Biagini , P. Bacchini , D. Donati , M. Manfrini , G. Bernini , S. Lari , Cancer 2003, 97, 3068. 12784343 10.1002/cncr.11456

[17] N. M. Marina , S. Smeland , S. S. Bielack , M. Bernstein , G. Jovic , M. D. Krailo , J. M. Hook , C. Arndt , H. van den Berg , B. Brennan , B. Brichard , K. L. B. Brown , T. Butterfass‐Bahloul , G. Calaminus , H. E. Daldrup‐Link , M. Eriksson , M. C. Gebhardt , H. Gelderblom , J. Gerss , R. Goldsby , A. Goorin , R. Gorlick , H. E. Grier , J. P. Hale , K. S. Hall , J. Hardes , D. S. Hawkins , K. Helmke , P. C. W. Hogendoorn , M. S. Isakoff , Lancet Oncol. 2016, 17, 1396. 27569442 10.1016/S1470-2045(16)30214-5 PMC5052459

[18] L. C. Sayles , M. R. Breese , A. L. Koehne , S. G. Leung , A. G. Lee , H.‐Y. Liu , A. Spillinger , A. T. Shah , B. Tanasa , K. Straessler , F. K. Hazard , S. L. Spunt , N. Marina , G. E. Kim , S.‐J. Cho , R. S. Avedian , D. G. Mohler , M.‐O. Kim , S. G. DuBois , D. S. Hawkins , E. A. Sweet‐Cordero , Cancer Discov. 2019, 9, 46. 30266815 10.1158/2159-8290.CD-17-1152 PMC7134333

[19] S. Xin , G. Wei , J. Bone Oncol. 2020, 21, 100281. 32140401 10.1016/j.jbo.2020.100281 PMC7047183

[20] K. Kawaguchi , K. Miyama , M. Endo , R. Bise , K. Kohashi , T. Hirose , A. Nabeshima , T. Fujiwara , Y. Matsumoto , Y. Oda , Y. Nakashima , npj Precis. Oncol 2024, 8, 16. 38253709 10.1038/s41698-024-00515-y PMC10803362

[21] Y. Huang , X. Zhai , T. Ma , M. Zhang , H. Yang , S. Zhang , J. Wang , W. Liu , X. Jin , W. W. Lu , X. Zhao , W. Hou , T. Sun , J. Shen , H. Pan , Y. Du , C.‐H. Yan , Adv. Mater. 2023, 35, 2300313. 10.1002/adma.202300313 36939167

[22] F. E. Freeman , P. Dosta , L. C. Shanley , N. Ramirez Tamez , C. J. Riojas Javelly , O. R. Mahon , D. J. Kelly , N. Artzi , Adv. Mater. 2023, 35, 2207877. 10.1002/adma.202207877 36994935

[23] S. Kortam , Z. Lu , H. Zreiqat , Commun. Mater. 2024, 5, 168.

[24] L. A. Aponte‐Tinao , M. A. Ayerza , J. I. Albergo , G. L. Farfalli , Clin. Orthop. Relat. Res. 2020, 478, 517. 32168064 10.1097/CORR.0000000000000806 PMC7145084

[25] M. Tsukasaki , H. Takayanagi , Nat. Rev. Immunol. 2019, 19, 626. 31186549 10.1038/s41577-019-0178-8

[26] T. Ikeuchi , N. M. Moutsopoulos , Bone 2022, 163, 116500. 35870792 10.1016/j.bone.2022.116500 PMC10448972

[27] I. E. Adamopoulos , Y. Choi , H. Takayanagi , Trends Immunol. 2025, 46, 192. 40011156 10.1016/j.it.2025.02.003 PMC11922659

[28] G. Cai , L. Ren , J. Yu , S. Jiang , G. Liu , S. Wu , B. Cheng , W. Li , J. Xia , Adv. Sci. 2024, 11, 2403786. 10.1002/advs.202403786 PMC11425865 38978324

[29] J.‐H. Lee , J. H. Park , J. H. Lee , H.‐H. Lee , J. C. Knowles , H.‐W. Kim , Matter 2022, 5, 3194.

[30] Z. Wan , X. Bai , X. Wang , X. Guo , X. Wang , M. Zhai , Y. Fu , Y. Liu , P. Zhang , X. Zhang , R. Yang , Y. Liu , L. Lv , Y. Zhou , Adv. Sci. 2024, 11, 2308986. 10.1002/advs.202308986 PMC11187922 38588510

[31] J. Zhang , D. Tong , H. Song , R. Ruan , Y. Sun , Y. Lin , J. Wang , L. Hou , J. Dai , J. Ding , H. Yang , Adv. Mater. 2022, 34, 2202044. 10.1002/adma.202202044 35785450

[32] R. Xu , H. Xie , X. Shen , J. Huang , H. Zhang , Y. Fu , P. Zhang , S. Guo , D. Wang , S. Li , K. Zheng , W. Sun , L. Liu , J. Cheng , H. Jiang , Adv. Sci. 2023, 10, 2303946. 10.1002/advs.202303946 PMC10754079 37897313

[33] B.‐R. Keum , H. J. Kim , J. Lee , M. Lee , S.‐H. Hong , H. K Chang , J.‐K. Han , S. Kim , D.‐G. Chang , G.‐H. Kim , Proc. Natl. Acad. Sci. USA 2024, 121, 2316871121. 10.1073/pnas.2316871121 PMC10895260 38346184

[34] C.‐C. Wu , H. C. Beird , J. Andrew Livingston , S. Advani , A. Mitra , S. Cao , A. Reuben , D. Ingram , W.‐L. Wang , Z. Ju , C. Hong Leung , H. Lin , Y. Zheng , J. Roszik , W. Wang , S. Patel , R. S. Benjamin , N. Somaiah , A. P. Conley , G. B. Mills , P. Hwu , R. Gorlick , A. Lazar , N. C. Daw , V. Lewis , P. A Futreal , Nat. Commun. 2020, 11, 1008. 32081846 10.1038/s41467-020-14646-w PMC7035358

[35] X. Wang , Z. Cui , Q. Jia , C. Hao , B. Wu , B. Wang , X. Shan , J. Gao , M. Du , Y. Li , J. Zhou , J. Liu , X. Zhang , Y. Fan , Adv. Funct. Mater. 2025, 2501317.

[36] J. Liao , R. Han , Y. Wu , Z. Qian , Bone Res. 2021, 9, 18. 33727543 10.1038/s41413-021-00139-z PMC7966774

[37] J. Long , W. Zhang , Y. Chen , B. Teng , B. Liu , H. Li , Z. Yao , D. Wang , L. Li , X.‐F. Yu , L. Qin , Y. Lai , Biomaterials 2021, 275, 120950. 34119886 10.1016/j.biomaterials.2021.120950

[38] J. Yin , S. Pan , X. Guo , Y. Gao , D. Zhu , Q. Yang , J. Gao , C. Zhang , Y. Chen , Nano Micro. Lett. 2021, 13, 30. 10.1007/s40820-020-00547-6 PMC8187678 34138204

[39] Q. Guan , T. Hu , L. Zhang , M. Yu , J. Niu , Z. Ding , P. Yu , G. Yuan , Z. An , J. Pei , Bioact. Mater. 2024, 40, 445. 39027327 10.1016/j.bioactmat.2024.06.026 PMC11255111

[40] Z. Wang , L. P. Nogueira , H. J. Haugen , I. C. M. Van Der Geest , P. C. de Almeida Rodrigues , D. Janssen , T. Bitter , J. J. J. P. van den Beucken , S. C. G. Leeuwenburgh , Bioact. Mater. 2022, 15, 120. 35386344 10.1016/j.bioactmat.2021.12.023 PMC8941180

[41] Y. Li , C. Liu , X. Cheng , J. Wang , Y. Pan , C. Liu , S. Zhang , X. Jian , Bioact. Mater. 2023, 27, 546. 37397628 10.1016/j.bioactmat.2023.04.020 PMC10313727

[42] Y. Zhao , Y. Xiong , Y. Zhao , Acc. Mater. Res. 2024, 5, 1532.

[43] Y.‐L. Yu , J.‐J. Wu , C.‐C. Lin , X. Qin , F. R. Tay , L. Miao , B.‐L. Tao , Y. Jiao , Mil. Med. Res. 2023, 10, 21. 37143145 10.1186/s40779-023-00454-y PMC10158155

[44] A. B. Asha , Y. Chen , R. Narain , Chem. Soc. Rev. 2021, 50, 11668. 34477190 10.1039/d1cs00658d

[45] S. W. Lee , K. S. Phillips , H. Gu , M. Kazemzadeh‐Narbat , D. Ren , Biomaterials 2021, 268, 120595. 33360301 10.1016/j.biomaterials.2020.120595

[46] C. Xu , S. Ivanovski , Nat. Rev. Bioeng. 2025, 3, 390.

[47] C. Li , C. Guo , V. Fitzpatrick , A. Ibrahim , M. J. Zwierstra , P. Hanna , A. Lechtig , A. Nazarian , S. J. Lin , D. L. Kaplan , Nat. Rev. Mater. 2020, 5, 61.

[48] R. C. Nordberg , B. J. Bielajew , T. Takahashi , S. Dai , J. C. Hu , K. A. Athanasiou , Nat. Rev. Rheumatol. 2024, 20, 323. 38740860 10.1038/s41584-024-01118-4 PMC11524031

[49] Y. Zhou , D. Yang , Q. Yang , X. Lv , W. Huang , Z. Zhou , Y. Wang , Z. Zhang , T. Yuan , X. Ding , L. Tang , J. Zhang , J. Yin , Y. Huang , W. Yu , Y. Wang , C. Zhou , Y. Su , A. He , Y. Sun , Z. Shen , B. Qian , W. Meng , J. Fei , Y. Yao , X. Pan , P. Chen , H. Hu , Nat. Commun. 2020, 11, 6322. 33303760 10.1038/s41467-020-20059-6 PMC7730477

[50] H. Tian , J. Cao , B. Li , E. C. Nice , H. Mao , Y. Zhang , C. Huang , Bone Res. 2023, 11, 11. 36849442 10.1038/s41413-023-00246-z PMC9971189

[51] A. Marchais , M. E. Marques da Costa , B. Job , R. Abbas , D. Drubay , S. Piperno‐Neumann , O. Fromigué , A. Gomez‐Brouchet , F. Redini , R. Droit , C. Lervat , N. Entz‐Werle , H. Pacquement , C. Devoldere , D. Cupissol , D. Bodet , V. Gandemer , M. Berger , P. Marec‐Berard , M. Jimenez , G. Vassal , B. Geoerger , L. Brugières , N. Gaspar , Cancer Res. 2022, 82, 974. 35078815 10.1158/0008-5472.CAN-20-4189

[52] J. Eigenbrood , N. Wong , P. Mallory , J. S. Pereira , D. Williams , D. W. Morris‐II , J. A. Beck , J. C. Cronk , C. M. Sayers , M. Mendez , L. Kaiser , J. Galindo , J. Singh , A. Cardamone , M. Pore , M. Kelly , A. K. LeBlanc , J. Cotter , R. N. Kaplan , T. A. McEachron , Cancer Res. 2025, 85, 2320. 40173049 10.1158/0008-5472.CAN-24-3723 PMC12170161

[53] W. Liu , H. Hu , Z. Shao , X. Lv , Z. Zhang , X. Deng , Q. Song , Y. Han , T. Guo , L. Xiong , B. Wang , Y. Zhang , Bone Res. 2023, 11, 4. 36596773 10.1038/s41413-022-00237-6 PMC9810605

[54] R. Liu , Y. Hu , T. Liu , Y. Wang , BMC Cancer 2021, 21, 1345. 34922489 10.1186/s12885-021-09042-6 PMC8684084

[55] W. Liu , X. Xie , Y. Qi , J. Wu , JAMA Netw. Open 2021, 4, 2119132. 10.1001/jamanetworkopen.2021.19132 PMC8335580 34342651

[56] H. Mu , Q. Zhang , D. Zuo , J. Wang , Y. Tao , Z. Li , X. He , H. Meng , H. Wang , J. Shen , M. Sun , Y. Jiang , W. Zhao , J. Han , M. Yang , Z. Wang , Y. Lv , Y. Yang , J. Xu , T. Zhang , L. Yang , J. Lin , F. Tang , R. Tang , H. Hu , Z. Cai , W. Sun , Y. Hua , Cell Rep. Med. 2025, 6, 101977. 39983717 10.1016/j.xcrm.2025.101977 PMC11970323

[57] K. Boye , A. Longhi , T. Guren , S. Lorenz , S. Næss , M. Pierini , I. Taksdal , I. Lobmaier , M. Cesari , A. Paioli , A. M. Løndalen , E. Setola , I. Hompland , L. A. Meza‐Zepeda , K. Sundby Hall , E. Palmerini , Cancer Immunol. Immunother. 2021, 70, 2617. 33580363 10.1007/s00262-021-02876-w PMC8360887

[58] C.‐Y. Sun , Z. Zhang , L. Tao , F.‐F. Xu , H.‐Y. Li , H‐Y. Zhang , W. Liu , Ann. Transl. Med. 2021, 9, 1447. 34733999 10.21037/atm-21-3928 PMC8506720

[59] C. Deng , Y. Xu , H. Chen , X. Zhu , L. Huang , Z. Chen , H. Xu , G. Song , J. Lu , W. Huang , R. Liu , Q. Tang , J. Wang , Cell Rep. 2024, 43, 113751. 38341855 10.1016/j.celrep.2024.113751

[60] C. Maccalli , G. Parmiani , S. Ferrone , Immunol. Invest. 2017, 46, 221. 28287848 10.1080/08820139.2017.1280051

[61] A. H. Long , S. L. Highfill , Y. Cui , J. P. Smith , A. J. Walker , S. Ramakrishna , R. El‐Etriby , S. Galli , M. G. Tsokos , R. J. Orentas , C. L. Mackall , Cancer Immunol. Res. 2016, 4, 869. 27549124 10.1158/2326-6066.CIR-15-0230 PMC5050151

[62] M. A. Riquelme , X. Wang , F. M. Acosta , J. Zhang , J. Chavez , S. Gu , P. Zhao , W. Xiong , N. Zhang , G. Li , S. Srinivasan , C. Ma , M. K. Rao , L.‐Z. Sun , N. Zhang , Z. An , J. X. Jiang , Cell Rep. 2024, 43, 114377. 38889005 10.1016/j.celrep.2024.114377 PMC11380445

[63] W. Qiao , K. H. M. Wong , J. Shen , W. Wang , J. Wu , J. Li , Z. Lin , Z. Chen , J. P. Matinlinna , Y. Zheng , S. Wu , X. Liu , K. P. Lai , Z. Chen , Y. W. Lam , K. M. C. Cheung , K. W. K. Yeung , Nat. Commun. 2021, 12, 2885. 34001887 10.1038/s41467-021-23005-2 PMC8128914

[64] J. Tuckermann , R. H. Adams , Nat. Rev. Rheumatol. 2021, 17, 608. 34480164 10.1038/s41584-021-00682-3 PMC7612477

[65] J. Wang , Y. Zhang , J. Cao , Y. Wang , N. Anwar , Z. Zhang , D. Zhang , Y. Ma , Y. Xiao , L. Xiao , X. Wang , Autophagy 2023, 19, 2409. 36858962 10.1080/15548627.2023.2186112 PMC10392742

[66] D. E. Place , R. K. S Malireddi , J. Kim , P. Vogel , M. Yamamoto , T.‐D. Kanneganti , Nat. Commun. 2021, 12, 496. 33479228 10.1038/s41467-020-20807-8 PMC7820603

[67] M. S. AlQranei , L. T. Senbanjo , H. Aljohani , T. Hamza , M. A. Chellaiah , BMC Immunol. 2021, 22, 23. 33765924 10.1186/s12865-021-00409-9 PMC7995782

[68] X. Yin , X. Teng , T. Ma , T. Yang , J. Zhang , M. Huo , W. Liu , Y. Yang , B. Yuan , H. Yu , W. Huang , Y. Wang , Cell Death Differ. 2022, 29, 2203. 35534547 10.1038/s41418-022-01010-2 PMC9613664

[69] L. Bai , Y. Liu , X. Zhang , P. Chen , R. Hang , Y. Xiao , J. Wang , C. Liu , Biomaterials 2023, 297, 122125. 37058900 10.1016/j.biomaterials.2023.122125

[70] X. Shen , X. Shen , B. Li , W. Zhu , Y. Fu , R. Xu , Y. Du , J. Cheng , H. Jiang , Bone 2021, 143, 115618. 32858254 10.1016/j.bone.2020.115618

[71] Y. Zhao , J. Ning , H. Teng , Y. Deng , M. Sheldon , L. Shi , C. Martinez , J. Zhang , A. Tian , Y. Sun , S. Nakagawa , F. Yao , H. Wang , L. Ma , Nat. Commun. 2024, 15, 2384. 38493144 10.1038/s41467-024-46602-3 PMC10944492

[72] A. Sawant , S. Ponnazhagan , Cancer Res. 2013, 73, 4606. 23887974 10.1158/0008-5472.CAN-13-0305 PMC3732563

[73] L. Zhang , K. H. Kwack , R. Thiyagarajan , K. K. Mullaney , N. A. Lamb , J. E. Bard , J. Sohn , K. L. Seldeen , Y. Arao , P. J. Blackshear , S. I. Abrams , B. R. Troen , K. L. Kirkwood , FASEB J. 2024, 38, 23338. 10.1096/fj.202301703R PMC11128769 38038723

[74] E. Seeman , Osteoporos. Int. 2003, 14, 2.12577179

[75] N. Tang , W.‐X. Song , J. Luo , R. C. Haydon , T.‐C. He , Clin. Orthop. Relat. Res. 2008, 466, 2114. 18563507 10.1007/s11999-008-0335-z PMC2492997

[76] M. L. Broadhead , J. C. M. Clark , D. E. Myers , C. R. Dass , P. F. M. Choong , Sarcoma 2011, 2011, 1. 10.1155/2011/959248 PMC3087974 21559216

[77] E. R. Wagner , G. Luther , G. Zhu , Q. Luo , Q. Shi , S. H. Kim , J.‐L. Gao , E. Huang , Y. Gao , K. Yang , L. Wang , C. Teven , X. Luo , X. Liu , M. Li , N. Hu , Y. Su , Y. Bi , B.‐C. He , N. Tang , J. Luo , L. Chen , G. Zuo , R. Rames , R. C. Haydon , H. H. Luu , T.‐C. He , Sarcoma 2011, 2011, 325238. 21437219 10.1155/2011/325238 PMC3061279

[78] K. Rickel , F. Fang , J. Tao , Bone 2017, 102, 69. 27760307 10.1016/j.bone.2016.10.017 PMC5393957

[79] T. Akiyama , C. R. Dass , P. F. M. Choong , Mol. Cancer Ther. 2008, 7, 3461. 19001431 10.1158/1535-7163.MCT-08-0530

[80] A. Alfranca , L. Martinez‐Cruzado , J. Tornin , A. Abarrategi , T. Amaral , E. de Alava , P. Menendez , J. Garcia‐Castro , R. Rodriguez , Cell. Mol. Life Sci. 2015, 72, 3097. 25935149 10.1007/s00018-015-1918-y PMC11113487

[81] S. R. Baglio , T. Lagerweij , M. Pérez‐Lanzón , X. D. Ho , N. Léveillé , S. A. Melo , A.‐M. Cleton‐Jansen , E. S. Jordanova , L. Roncuzzi , M. Greco , M. A. J. van Eijndhoven , G. Grisendi , M. Dominici , R. Bonafede , S. M. Lougheed , T. D. de Gruijl , N. Zini , S. Cervo , A. Steffan , V. Canzonieri , A. Martson , K. Maasalu , S. Köks , T. Wurdinger , N. Baldini , D. M. Pegtel , Clin. Cancer Res. 2017, 23, 3721. 28053020 10.1158/1078-0432.CCR-16-2726

[82] S. Avnet , G. Di Pompo , T. Chano , C. Errani , A. Ibrahim‐Hashim , R. J. Gillies , D. M. Donati , N. Baldini , Int. J. Cancer 2017, 140, 1331. 27888521 10.1002/ijc.30540 PMC5272857

[83] R. Rubio , A. Abarrategi , J. Garcia‐Castro , L. Martinez‐Cruzado , C. Suarez , J. Tornin , L. Santos , A. Astudillo , I. Colmenero , F. Mulero , M. Rosu‐Myles , P. Menendez , R. Rodriguez , Stem Cells 2014, 32, 1136. 24446210 10.1002/stem.1647

[84] W.‐t. Xu , Z.‐y. Bian , Q.‐m. Fan , G. Li , T.‐t. Tang , Cancer Lett. 2009, 281, 32. 19342158 10.1016/j.canlet.2009.02.022

[85] C. Massaro , H. N. Sensoy , M. Mulders , C. De Schrijver , C. Gómez‐Martín , J. Simon Nieto , T. Lagerweij , A. Atmopawiro , J. Pérez‐Boza , M. Bebelman , L. Bosch , S. Foderaro , M. Neves Ferreira , M. A. J. Van Eijndhoven , J. R. T. Van Weering , C. Dell'Aversana , L. Altucci , C. D. Savci‐Heijink , N. W. C. J. Van De Donk , C. Giorgio , L. Brandolini , M. Allegretti , D. M. Pegtel , S. R. Baglio , Clin. Cancer Res. 2024, 30, 4714. 39115426 10.1158/1078-0432.CCR-23-4097

[86] F. Tang , Y. Tie , T.‐X. Lan , J.‐Y. Yang , W.‐Q. Hong , S.‐Y. Chen , H.‐H. Shi , L.‐Q. Li , H. Zeng , L. Min , Y.‐Q. Wei , C.‐Q. Tu , X.‐W. Wei , Adv. Sci. 2023, 10, 2207518. 10.1002/advs.202207518 PMC10558698 37585564

[87] J. A. Krall , F. Reinhardt , O. A. Mercury , D. R. Pattabiraman , M. W. Brooks , M. Dougan , A. W. Lambert , B. Bierie , H. L. Ploegh , S. K. Dougan , R. A. Weinberg , Sci. Transl. Med. 2018, 10, aan3464. 10.1126/scitranslmed.aan3464 PMC6364295 29643230

[88] S. Smeland , S. S. Bielack , J. Whelan , M. Bernstein , P. Hogendoorn , M. D. Krailo , R. Gorlick , K. A. Janeway , F. C. Ingleby , J. Anninga , I. Antal , C. Arndt , K. L. B. Brown , T. Butterfass‐Bahloul , G. Calaminus , M. Capra , C. Dhooge , M. Eriksson , A. M. Flanagan , G. Friedel , M. C. Gebhardt , H. Gelderblom , R. Goldsby , H. E. Grier , R. Grimer , D. S. Hawkins , S. Hecker‐Nolting , K. Sundby Hall , M. S. Isakoff , G. Jovic , Eur. J. Cancer 2019, 109, 36. 30685685 10.1016/j.ejca.2018.11.027 PMC6506906

[89] X. Cheng , H. Zhang , A. Hamad , H. Huang , A. Tsung , Semin. Cancer Biol. 2022, 86, 408. 35066156 10.1016/j.semcancer.2022.01.006 PMC11770836

[90] M. P. Kallis , C. Maloney , B. Blank , S. Z. Soffer , M. Symons , B. M. Steinberg , J. Transl. Med. 2020, 18, 183. 32354335 10.1186/s12967-020-02348-2 PMC7193344

[91] M.‐F. Heymann , F. Lézot , D. Heymann , Cell. Immunol. 2019, 343, 103711. 29117898 10.1016/j.cellimm.2017.10.011

[92] X. Huang , Y. Liu , W. Liang , K. Luo , Y. Qin , F. Li , T. Xie , H. Qin , J. He , Q. Wei , BMC Cancer 2022, 22, 1370. 36585638 10.1186/s12885-022-10477-8 PMC9805258

[93] C. Ma , R. Yu , J. Li , J. Guo , J. Xu , X. Wang , P. Liu , J. Surg. Oncol. 2022, 125, 754. 34811745 10.1002/jso.26757

[94] F. Tang , Y. Tie , C. Tu , X. Wei , Clin. Transl. Med. 2020, 10, 199. 32508035 10.1002/ctm2.24 PMC7240866

[95] S. Yu , X. Yao , Mol. Cancer 2024, 23, 192. 39245737 10.1186/s12943-024-02105-9 PMC11382402

[96] M. P. Kallis , C. Maloney , M. Edelman , S. Z. Soffer , M. Symons , B. M. Steinberg , Cancer Res. 2020, 80, PR13. 10.1158/1535-7163.MCT-19-0903 32371577

[97] L. K. Chim , I. L. Williams , C. J. Bashor , A. G. Mikos , Biomaterials 2023, 296, 122076. 36931102 10.1016/j.biomaterials.2023.122076 PMC11132719

[98] Z. Wang , B. Li , S. Li , W. Lin , Z. Wang , S. Wang , W. Chen , W. Shi , T. Chen , H. Zhou , E. Yinwang , W. Zhang , H. Mou , X. Chai , J. Zhang , Z. Lu , Z. Ye , Nat. Commun. 2022, 13, 6308. 36274066 10.1038/s41467-022-34064-4 PMC9588779

[99] J. A. Ligon , W. Choi , G. Cojocaru , W. Fu , E. H.‐C. Hsiue , T. F. Oke , N. Siegel , M. H. Fong , B. Ladle , C. A. Pratilas , C. D. Morris , A. Levin , D. S. Rhee , C. F. Meyer , A. J. Tam , R. Blosser , E. D. Thompson , A. Suru , D. Mcconkey , F. Housseau , R. Anders , D. M. Pardoll , N. Llosa , J. ImmunoTher. Cancer 2021, 9, 001772. 10.1136/jitc-2020-001772 PMC8144029 34021032

[100] C. Chen , L. Xie , T. Ren , Y. Huang , J. Xu , W. Guo , Cancer Lett. 2021, 500, 1. 33359211 10.1016/j.canlet.2020.12.024

[101] M. Panagi , P. Pilavaki , A. Constantinidou , T. Stylianopoulos , Theranostics 2022, 12, 6106. 36168619 10.7150/thno.72800 PMC9475460

[102] F. Jafari , S. Javdansirat , S. Sanaie , A. Naseri , A. Shamekh , D. Rostamzadeh , S. Dolati , Ann. Diagn. Pathol. 2020, 49, 151654. 33130384 10.1016/j.anndiagpath.2020.151654

[103] L. Ma , X. Feng , H. Liang , K. Wang , Y. Song , L. Tan , B. Wang , R. Luo , Z. Liao , G. Li , X. Liu , S. Wu , C. Yang , Mater. Today 2020, 36, 48.

[104] C. P. Carty , I. C. Dickinson , M. C. Watts , R. W. Crawford , P. Steadman , Knee 2009, 16, 405. 19269182 10.1016/j.knee.2009.02.006

[105] A. Al Subaie , E. Emami , I. Tamimi , M. Laurenti , H. Eimar , M.‐N. Abdallah , F. Tamimi , J. Clin. Periodontol. 2016, 43, 193. 26725944 10.1111/jcpe.12506

[106] B. A. Lindsey , J. E. Markel , E. S. Kleinerman , Rheumatol. Ther. 2017, 4, 25. 27933467 10.1007/s40744-016-0050-2 PMC5443719

[107] M. Kansara , M. W. Teng , M. J. Smyth , D. M. Thomas , Nat. Rev. Cancer 2014, 14, 722. 25319867 10.1038/nrc3838

[108] F. Loi , L. A. Córdova , R. Zhang , J. Pajarinen , T.‐H. Lin , S. B. Goodman , Z. Yao , Stem Cell Res. Ther. 2016, 7, 15. 26801095 10.1186/s13287-016-0276-5 PMC4724110

[109] N. J. Horwood , Clin. Rev. Allergy Immunol. 2016, 51, 79. 26498771 10.1007/s12016-015-8519-2

[110] H. Takayanagi , Nat. Rev. Immunol. 2007, 7, 292. 17380158 10.1038/nri2062

[111] Z. Zhao , Y. Du , K. Yan , L. Zhang , Q. Guo , FASEB J. 2024, 38, 23554. 10.1096/fj.202301508RRR 38588175

[112] G. Yu , P. G. Corn , C. S. L. Mak , X. Liang , M. Zhang , P. Troncoso , J. H. Song , S.‐C. Lin , X. Song , J. Liu , J. Zhang , C. J. Logothetis , M. P. Melancon , T. Panaretakis , G. Wang , S.‐H. Lin , Proc. Natl. Acad. Sci. USA 2024, 121, 2402903121. 10.1073/pnas.2402903121 PMC11331113 39102549

[113] D. T. Ammons , L. S. Hopkins , K. E. Cronise , J. Kurihara , D. P. Regan , S. Dow , Commun. Biol. 2024, 7, 496. 38658617 10.1038/s42003-024-06182-w PMC11043452

[114] R.‐Y. Ma , A. Black , B.‐Z. Qian , Trends Immunol. 2022, 43, 546. 35690521 10.1016/j.it.2022.04.008

[115] Y. Cheng , F. Bai , X. Ren , R. Sun , X. Guo , W. Liu , B. Wang , Y. Yang , X. Zhang , Y. Xu , C. Li , X. Yang , L. Gao , C. Ma , X. Li , X. Liang , Cancer Res. 2022, 82, 1603. 35135809 10.1158/0008-5472.CAN-21-0003

[116] B. K. Nirala , T. D. Patel , L. Kurenbekova , R. Shuck , A. Dasgupta , N. Rainusso , C. Coarfa , J. T. Yustein , JCI Insight 2023, 8, 164947. 10.1172/jci.insight.164947 PMC10371352 37279073

[117] Z. Wang , H. Wu , Y. Chen , H. Chen , W. Yuan , X. Wang , J. Oncol. 2021, 2021, 4836292. 34335756 10.1155/2021/4836292 PMC8321719

[118] J. Li , C. Zhao , Y. Li , J. Wen , S. Wang , D. Wang , H. Dong , D. Wang , Y. Zhao , X. Wang , X. He , J. Qin , Cancer Lett. 2022, 528, 1. 34952143 10.1016/j.canlet.2021.12.023

[119] M.‐F. Heymann , K. Schiavone , D. Heymann , Br. J. Pharmacol. 2021, 178, 1955. 31975481 10.1111/bph.14999

[120] P. M. Giraldo‐Osorno , K. Wirsig , F. Asa'ad , O. Omar , M. Trobos , A. Bernhardt , A. Palmquist , Acta Biomater. 2024, 186, 141. 39142531 10.1016/j.actbio.2024.08.005

[121] D. Andreev , K. Kachler , G. Schett , A. Bozec , Bone 2022, 162, 116468. 35688359 10.1016/j.bone.2022.116468

[122] J. Ping , C. Zhou , Y. Dong , X. Wu , X. Huang , B. Sun , B. Zeng , F. Xu , W. Liang , Mol. Immunol. 2021, 138, 110. 34392109 10.1016/j.molimm.2021.08.003

[123] X. Huang , L. Wang , H. Guo , W. Zhang , Bioact. Mater. 2023, 23, 69. 36406251 10.1016/j.bioactmat.2022.09.027 PMC9650013

[124] J. H. Rannikko , M. Hollmén , Br. J. Cancer 2024, 131, 627. 38831013 10.1038/s41416-024-02715-6 PMC11333586

[125] E. P. Young , C. A. Johnson , A. G. Lee , C. R. Schott , E. A. Sweet‐Cordero , Cancer Immunol. Res. 2023, 11, B036.

[126] A. R. Cillo , E. Mukherjee , N. G. Bailey , S. Onkar , J. Daley , C. Salgado , X. Li , D. Liu , S. Ranganathan , M. Burgess , J. Sembrat , K. Weiss , R. Watters , T. C. Bruno , D. A. A. Vignali , K. M. Bailey , Clin. Cancer Res. 2022, 28, 4968. 36074145 10.1158/1078-0432.CCR-22-1471 PMC9669190

[127] M. Liu , R. S. O'Connor , S. Trefely , K. Graham , N. W. Snyder , G. L. Beatty , Nat. Immunol. 2019, 20, 265. 30664738 10.1038/s41590-018-0292-y PMC6380920

[128] Z. Dongye , J. Li , Y. Wu , Br. J. Cancer 2022, 127, 1584. 35902641 10.1038/s41416-022-01876-6 PMC9333350

[129] M. Kortylewski , M. Kujawski , A. Herrmann , C. Yang , L. Wang , Y. Liu , R. Salcedo , H. Yu , Cancer Res. 2009, 69, 2497. 19258507 10.1158/0008-5472.CAN-08-3031 PMC2657819

[130] M. J. Sweet , D. Ramnath , A. Singhal , R. Kapetanovic , Nat. Rev. Immunol. 2025, 25, 92. 39294278 10.1038/s41577-024-01080-y

[131] X. Wang , S. Zhang , D. Xue , D. Neculai , J. Zhang , Trends Endocrinol. Metab. 2025, 36, 660. 39304355 10.1016/j.tem.2024.08.009

[132] A. Sindrilaru , T. Peters , S. Wieschalka , C. Baican , A. Baican , H. Peter , A. Hainzl , S. Schatz , Y. Qi , A. Schlecht , J. M. Weiss , M. Wlaschek , C. Sunderkötter , K. Scharffetter‐Kochanek , J. Clin. Invest. 2011, 121, 985. 21317534 10.1172/JCI44490 PMC3049372

[133] S. Zanganeh , G. Hutter , R. Spitler , O. Lenkov , M. Mahmoudi , A. Shaw , J. S. Pajarinen , H. Nejadnik , S. Goodman , M. Moseley , L. M. Coussens , H. E. Daldrup‐Link , Nat. Nanotechnol. 2016, 11, 986. 27668795 10.1038/nnano.2016.168 PMC5198777

[134] Y. Xue , X. Yan , D. Li , S. Dong , Y. Ping , Nat. Commun. 2024, 15, 2270. 38491004 10.1038/s41467-024-46210-1 PMC10943244

[135] T. Tufan , G. Comertpay , A. Villani , G. M. Nelson , M. Terekhova , S. Kelley , P. Zakharov , R. M. Ellison , O. Shpynov , M. Raymond , J. Sun , Y. Chen , E. Bockelmann , M. Stremska , L. W. Peterson , L. Boeckaerts , S. R. Goldman , J. I Etchegaray , M. N. Artyomov , F. Peri , K. S. Ravichandran , Nature 2024, 628, 408. 38480883 10.1038/s41586-024-07172-y PMC12573440

[136] Y. Sun , C. Zhang , Q. Fang , W. Zhang , W. Liu , J. Transl. Med. 2023, 21, 99. 36759884 10.1186/s12967-023-03961-7 PMC9912612

[137] S. Rajan , S. Zaccaria , M. V. Cannon , M. Cam , A. C. Gross , B. J. Raphael , R. D. Roberts , Cancer Res. Commun. 2023, 3, 564. 37066022 10.1158/2767-9764.CRC-22-0348 PMC10093779

[138] D. D. Truong , C. Weistuch , K. A. Murgas , P. Admane , B. L. King , J. Chauviere Lee , S.‐E. Lamhamedi‐Cherradi , J. Swaminathan , N. C. Daw , N. Gordon , V. Gopalakrishnan , R. G. Gorlick , N. Somaiah , J. O. Deasy , A. G. Mikos , A. Tannenbaum , J. Ludwig , Clin. Cancer Res. 2024, 30, 3259. 38775859 10.1158/1078-0432.CCR-24-0563 PMC11293971

[139] Z. Li , X. Lai , S. Fu , L. Ren , H. Cai , H. Zhang , Z. Gu , X. Ma , K. Luo , Adv. Sci. 2022, 9, 2201734. 10.1002/advs.202201734 PMC9353475 35652198

[140] C. Huang , B. Lin , C. Chen , H. Wang , X. Lin , J. Liu , Q. Ren , J. Tao , P. Zhao , Y. Xu , Adv. Mater. 2022, 34, 2207593. 10.1002/adma.202207593 36245299

[141] P. Meier , A. J. Legrand , D. Adam , J. Silke , Nat. Rev. Cancer 2024, 24, 299. 38454135 10.1038/s41568-024-00674-x

[142] L. Galluzzi , E. Guilbaud , D. Schmidt , G. Kroemer , F. M. Marincola , Nat. Rev. Drug Discovery 2024, 23, 445. 38622310 10.1038/s41573-024-00920-9 PMC11153000

[143] C. Zhao , C. Wang , W. Shan , Z. Wang , X. Chen , H. Deng , Acc. Chem. Res. 2024, 57, 905. 38417027 10.1021/acs.accounts.3c00771

[144] C. Li , L. Tu , Y. Xu , M. Li , J. Du , P. J. Stang , Y. Sun , Y. Sun , Angew. Chem., Int. Ed. 2024, 63, 202406392. 10.1002/anie.202406392 38775364

[145] J. Huang , J. Yang , Y. Yang , X. Lu , J. Xu , S. Lu , H. Pan , W. Zhou , W. Li , S. Chen , Adv. Sci. 2025, 12, 2415227. 10.1002/advs.202415227 PMC12061326 40052211

[146] L. Cheng , Y. Wang , Y. Zhang , Trends Cancer 2025, 11, 376. 39986988 10.1016/j.trecan.2025.01.012

[147] J. Zhang , T. Gai , J. Wang , Y. Wu , S.‐M. Zeng , D. Zhao , W. Li , Adv. Sci. 2025, 12, 2503299. 10.1002/advs.202503299 PMC12245015 40184610

[148] W. Xiong , Z. Cheng , H. Chen , H. Liang , M. Wang , Y. Chen , J. Ying , Y. Cai , J. Chai , K. Dou , W. Zheng , S. Zheng , L. Zhao , Adv. Funct. Mater. 2024, 34, 2410841.

[149] W. Zhong , W. Yuan , Y. Chen , Z. Ma , M. Ma , B. S. N. Tan , J. Yang , Y. Zhao , Angew. Chem., Int. Ed. 2024, 63, 202401250. 10.1002/anie.202401250 38576254

[150] M. Wang , F. Xue , L. An , D. Wu , S. Sha , G. Huang , Q. Tian , Adv. Funct. Mater. 2024, 34, 2311853.

[151] J. Liang , X. Qiao , L. Qiu , H. Xu , H. Xiang , H. Ding , Y. Chen , Adv. Sci. 2024, 11, 2305392. 10.1002/advs.202305392 PMC10797440 38041509

[152] M. A. Schneider , L. Heeb , M. M. Beffinger , S. Pantelyushin , M. Linecker , L. Roth , K. Lehmann , U. Ungethüm , S. Kobold , R. Graf , M. Van Den Broek , J. Vom Berg , A. Gupta , P.‐A. Clavien , Sci. Transl. Med. 2021, 13, abc8188. 10.1126/scitranslmed.abc8188 34524861

[153] S. C. Wei , C. R. Duffy , J. P. Allison , Cancer Discov. 2018, 8, 1069. 30115704 10.1158/2159-8290.CD-18-0367

[154] P. Sharma , S. Goswami , D. Raychaudhuri , B. A. Siddiqui , P. Singh , A. Nagarajan , J. Liu , S. K. Subudhi , C. Poon , K. L. Gant , S. M. Herbrich , S. Anandhan , S. Islam , M. Amit , G. Anandappa , J. P. Allison , Cell 2023, 186, 1652. 37059068 10.1016/j.cell.2023.03.006

[155] M. R. Pitter , W. Zou , Cancer Res. 2021, 81, 5141. 34654698 10.1158/0008-5472.CAN-21-2926 PMC8974405

[156] A. Kennedy , E. Waters , B. Rowshanravan , C. Hinze , C. Williams , D. Janman , T. A. Fox , C. Booth , A. M. Pesenacker , N. Halliday , B. Soskic , S. Kaur , O. S. Qureshi , E. C. Morris , S. Ikemizu , C. Paluch , J. Huo , S. J. Davis , E. Boucrot , L. S. K. Walker , D. M. Sansom , Nat. Immunol. 2022, 23, 1365. 35999394 10.1038/s41590-022-01289-w PMC9477731

[157] F. Marangoni , A. Zhakyp , M. Corsini , S. N. Geels , E. Carrizosa , M. Thelen , V. Mani , J. N. Prüßmann , R. D. Warner , A. J. Ozga , M. Di Pilato , S. Othy , T. R. Mempel , Cell 2021, 184, 3998. 34157302 10.1016/j.cell.2021.05.027 PMC8664158

[158] J. W. Kjeldsen , C. L. Lorentzen , E. Martinenaite , E. Ellebaek , M. Donia , R. B. Holmstroem , T. W. Klausen , C. O. Madsen , S. M. Ahmed , S. E. Weis‐Banke , M. O. Holmström , H. W. Hendel , E. Ehrnrooth , M.‐B. Zocca , A. W. Pedersen , M. H. Andersen , I. M. Svane , Nat. Med. 2021, 27, 2212. 34887574 10.1038/s41591-021-01544-x PMC8904254

[159] N. K. Verma , B. H. S. Wong , Z. S. Poh , A. Udayakumar , R. Verma , R. K. J. Goh , S. P. Duggan , V. G. Shelat , K. G Chandy , N. F. Grigoropoulos , eBioMedicine 2022, 83, 104216. 35986950 10.1016/j.ebiom.2022.104216 PMC9403334

[160] D. B. Johnson , C. A. Nebhan , J. J. Moslehi , J. M. Balko , Nat. Rev. Clin. Oncol. 2022, 19, 254. 35082367 10.1038/s41571-022-00600-w PMC8790946

[161] P. C. Johnson , J. F. Gainor , R. J. Sullivan , D. L. Longo , B. Chabner , N. Engl. J. Med. 2023, 388, 1529. 37075146 10.1056/NEJMsb2300232

[162] Y. Wen , F. Tang , C. Tu , F. Hornicek , Z. Duan , L. Min , Cancer Lett. 2022, 547, 215887. 35995141 10.1016/j.canlet.2022.215887

[163] P. Thanindratarn , D. C. Dean , S. D. Nelson , F. J. Hornicek , Z. Duan , J. Bone Oncol. 2019, 15, 100221. 30775238 10.1016/j.jbo.2019.100221 PMC6365405

[164] C. Feng , Y. Jiang , T. Wang , D. Tian , C. Shen , Y. Wang , H. Qian , Coord. Chem. Rev. 2023, 493, 215315.

[165] Y. Zhang , J. Cui , K‐Y. Chen , S. H. Kuo , J. Sharma , R. Bhatta , Z. Liu , A. Ellis‐Mohr , F. An , J. Li , Q. Chen , K. D. Foss , H. Wang , Y. Li , A. M. Mccoy , G. W. Lau , Q. Cao , Sci. Adv. 2023, 9, adg7397. 10.1126/sciadv.adg7397 PMC10162669 37146142

[166] S. Talebian , B. Mendes , J. Conniot , S. Farajikhah , F. Dehghani , Z. Li , D. Bitoque , G. Silva , S. Naficy , J. Conde , G. G. Wallace , Adv. Sci. 2023, 10, 2207603. 10.1002/advs.202207603 PMC10131825 36782094

[167] M. Veletić , E. H. Apu , M. Simić , J. Bergsland , I. Balasingham , C. H. Contag , N. Ashammakhi , Chem. Rev. 2022, 122, 16329. 35981266 10.1021/acs.chemrev.2c00005

[168] Q. Zhang , R.‐L. Pan , H. Wang , J.‐J. Wang , S.‐H. Lu , M. Zhang , Nano Lett. 2024, 24, 8257. 38920296 10.1021/acs.nanolett.4c01101 PMC11247543

[169] Y. Zhu , H. Liang , X. Liu , J. Wu , C. Yang , T. M. Wong , K. Y. H. Kwan , K. M. C. Cheung , S. Wu , K. W. K. Yeung , Sci. Adv. 2021, 7, abf6654. 10.1126/sciadv.abf6654 PMC11060047 33811079

[170] B. Zhang , Y. Su , J. Zhou , Y. Zheng , D. Zhu , Adv. Sci. 2021, 8, 2100446. 10.1002/advs.202100446 PMC8373114 34117732

[171] Z. Dong , X. Ke , S. Tang , S. Wu , W. Wu , X. Chen , J. Yang , J. Xie , J. Luo , J. Li , Chem. Mater. 2021, 33, 7994.

[172] C. Li , W. Zhang , Y. Nie , X. Du , C. Huang , L. Li , J. Long , X. Wang , W. Tong , L. Qin , Y. Lai , Adv. Mater. 2024, 36, 2308875. 10.1002/adma.202308875 38091500

[173] G. Gong , H. Huang , Z. Tong , Y. Zheng , D. Bian , Y. Zhang , Biomaterials 2025, 320, 123263. 40132359 10.1016/j.biomaterials.2025.123263

[174] M. Li , T. Wei , H. Liu , Z. Wu , J. Zhou , J. Jiang , P. He , Y. Zhang , Y. Zheng , Adv. Funct. Mater. 2025, 10.1002/adfm.202507822.

[175] C. Li , C. Li , Z. Ma , H. Chen , H. Ruan , L. Deng , J. Wang , W. Cui , Bioact. Mater. 2023, 19, 474. 35574049 10.1016/j.bioactmat.2022.04.028 PMC9079115

[176] X. Liu , H. Zhang , S. Guan , J. Tan , K. W. K. Yeung , L. Ouyang , X. Liu , ACS Nano 2025, 19, 14954. 40197016 10.1021/acsnano.5c00721

[177] H. Wang , Y. Chen , R. Wei , J. Zhang , J. Zhu , W. Wang , Z. Wang , Z. Wupur , Y. Li , H. Meng , Adv. Mater. 2024, 36, 2309591. 10.1002/adma.202309591 38113900

[178] C.‐H. Lee , W.‐Y. Huang , K.‐Y. Lee , C.‐H. Kuan , T.‐C. Wu , J.‐S. Sun , T.‐W. Wang , Chem. Eng. J. 2024, 486, 150236.

[179] H.‐Y. Gu , J.‐X. Fan , P. Bao , W.‐Q. Qu , Z.‐B. Hu , B.‐W. Qi , X.‐Z. Zhang , A.‐X. Yu , Adv. Funct. Mater. 2024, 34, 2311015.

[180] Y. Ma , P. Lai , Z. Sha , B. Li , J. Wu , X. Zhou , C. He , X. Ma , Bioact. Mater. 2025, 47, 83. 39897587 10.1016/j.bioactmat.2025.01.006 PMC11783017

[181] X. Wang , X. Guo , H. Ren , X. Song , L. Chen , L. Yu , J. Ren , Y. Chen , Adv. Mater. 2025, 37, 2415814. 10.1002/adma.202415814 39726343

[182] Z. Yan , Y. Deng , L. Huang , J. Zeng , D. Wang , Z. Tong , Q. Fan , W. Tan , J. Yan , X. Zang , S. Chen , J. Nanobiotechnol. 2025, 23, 286. 10.1186/s12951-025-03253-w PMC11983740 40205459

[183] L. Chen , X. Wu , D. Li , S. Jia , J. Yu , B. Li , J. Li , L. Bai , S. Guan , Chem. Eng. J. 2025, 509, 161350.

[184] G. Kroemer , C. Galassi , L. Zitvogel , L. Galluzzi , Nat. Immunol. 2022, 23, 487. 35145297 10.1038/s41590-022-01132-2

[185] G. Kroemer , L. Galluzzi , O. Kepp , L. Zitvogel , Annu. Rev. Immunol. 2013, 31, 51. 23157435 10.1146/annurev-immunol-032712-100008

[186] Z. Zhang , Z. Pan , Q. Li , Q. Huang , L. Shi , Y. Liu , Sci. Adv. 2024, 10, adk0716. 10.1126/sciadv.adk0716 PMC10849581 38324678

[187] Q. Meng , B. Ding , P. a. Ma , J. Lin , Acc. Chem. Res. 2025, 58, 1210. 40179239 10.1021/acs.accounts.4c00843

[188] J. Liu , Z. Li , D. Zhao , X. Feng , C. Wang , D. Li , J. Ding , Mater. Des. 2021, 202, 109465.

[189] X. Chu , B. Mi , Y. Xiong , R. Wang , T. Liu , L. Hu , C. Yan , R. Zeng , J. Lin , H. Fu , G. Liu , K. Zhang , L. Bian , Biomaterials 2025, 312, 122714. 39079462 10.1016/j.biomaterials.2024.122714

[190] J. Ge , N. Yang , Y. Yang , H. Yu , X. Yang , Y. Wang , T. Wang , S. Cheng , Y. Wang , Z. Han , Y. Teng , J. Zou , H. Yang , L. Cheng , Bioact. Mater. 2023, 25, 73. 36733928 10.1016/j.bioactmat.2023.01.008 PMC9883145

[191] L. Ma , J. Zhou , Q. Wu , G. Luo , M. Zhao , G. Zhong , Y. Zheng , X. Meng , S. Cheng , Y. Zhang , Biomaterials 2023, 301, 122236. 37506512 10.1016/j.biomaterials.2023.122236

[192] N. A. Pechnikova , A. Aggeli , A. A. Latypova , A. V. Iaremenko , K. Domvri , I. V. Zubarev , C. Liu , A. V. Yaremenko , Adv. Funct. Mater. 2025, 35, 2416813.

[193] C. Yang , Y. Luo , H. Shen , M. Ge , J. Tang , Q. Wang , H. Lin , J. Shi , X. Zhang , Nat. Commun. 2022, 13, 4866. 35982036 10.1038/s41467-022-32405-x PMC9388665

[194] C. Zhang , P. Ma , A. Qin , L. Wang , K. Dai , Y. Liu , J. Zhao , Z. Lu , Research 2023, 6, 0220. 39902178 10.34133/research.0220 PMC11789687

[195] Y. Yuan , H. Li , Y. Song , D. Zhang , Z. Wang , X. Yi , B. Qi , X. Zhang , P. Jiang , A. Yu , Adv. Mater. 2024, 36, 2408473. 10.1002/adma.202408473 39212208

[196] D. J. Irvine , Nat. Rev. Mater. 2016, 1, 15008.

[197] C. He , L. Yu , H. Yao , Y. Chen , Y. Hao , Adv. Funct. Mater. 2021, 31, 2006214.

[198] C. A. Lahr , M. Landgraf , F. Wagner , A. Cipitria , I. Moreno‐Jiménez , O. Bas , B. Schmutz , C. Meinert , A. D. S. Cavalcanti , T. Mashimo , Y. Miyasaka , B. M. Holzapfel , A. Shafiee , J. A. Mcgovern , D. W. Hutmacher , Bone 2022, 158, 116018. 34023543 10.1016/j.bone.2021.116018

[199] L. Zhang , J. Liu , H. Zhang , Y. Qian , L. Zhang , W. Wang , In Vivo 2024, 38, 2665. 39477440 10.21873/invivo.13743 PMC11535942

[200] S. Jarvis , E. Koumadoraki , N. Madouros , S. Sharif , A. Saleem , S. Khan , Cancer Treat. Res. Commun. 2021, 27, 100307. 33453605 10.1016/j.ctarc.2021.100307

[201] E. A. Ogunnaike , A. Valdivia , M. Yazdimamaghani , E. Leon , S. Nandi , H. Hudson , H. Du , S. Khagi , Z. Gu , B. Savoldo , F. S. Ligler , S. Hingtgen , G. Dotti , Sci. Adv. 2021, 7, abg5841. 10.1126/sciadv.abg5841 PMC8494441 34613775

[202] Y. Lu , A. Liu , S. Jin , J. Dai , Y. Yu , P. Wen , Y. Zheng , D. Xia , Adv. Mater. 2025, 37, 2410589. 10.1002/adma.202410589 39564691

[203] J. Li , S. Liao , Y. Wu , J. Bi , Y. Han , Y. Zhang , M. Xu , W. Bi , Nano Today 2023, 50, 101877;

[204] H. Wei , J. Cui , K. Lin , J. Xie , X. Wang , Bone Res. 2022, 10, 17. 35197462 10.1038/s41413-021-00180-y PMC8866424

[205] J. Zhang , Y. Zhuang , R. Sheng , H. Tomás , J. Rodrigues , G. Yuan , X. Wang , K. Lin , Mater. Horiz. 2024, 11, 12. 37818593 10.1039/d3mh01260c

[206] Y. Bian , K. Zhao , T. Hu , C. Tan , R. Liang , X. Weng , Adv. Sci. 2024, 11, 2403791. 10.1002/advs.202403791 PMC11434235 38958509

[207] H. Lin , X. Jin , Y. Cao , R. Ruan , C. Liu , S. Huang , J. Xu , J. Ding , H. Yang , J. Zhang , Adv. Funct. Mater. 2024, 10.1002/adfm.202421470.

[208] S. C. Casey , L. Tong , Y. Li , R. Do , S. Walz , K. N. Fitzgerald , A. M. Gouw , V. Baylot , I. Gütgemann , M. Eilers , D. W. Felsher , Science 2016, 352, 227. 26966191 10.1126/science.aac9935 PMC4940030

[209] K. Jiang , Q. Zhang , Y. Fan , J. Li , J. Zhang , W. Wang , J. Fan , Y. Guo , S. Liu , D. Hao , Y. Wang , L. Wang , L. Shan , Cell Death Discov. 2022, 8, 117. 35292660 10.1038/s41420-022-00923-8 PMC8924240

[210] Z. Liao , M. Li , G. Wen , K. Wang , D. Yao , E. Chen , Y. Liang , T. Xing , K. Su , C. Liang , Z. Che , Q. Ning , J. Tang , W. Yan , Y. Li , L. Huang , npj Precis. Oncol 2023, 7, 62. 37386055 10.1038/s41698-023-00415-7 PMC10310742

[211] G. Li , S. Liu , Y. Chen , J. Zhao , H. Xu , J. Weng , F. Yu , A. Xiong , A. Udduttula , D. Wang , P. Liu , Y. Chen , H. Zeng , Nat. Commun. 2023, 14, 3159. 37258510 10.1038/s41467-023-38597-0 PMC10232438

[212] F. Jahanmard , A. Khodaei , J. Flapper , O. Dogan , K. Roohi , P. Taheri , H. Weinans , G. Storm , M. Croes , E. Mastrobattista , S. Amin Yavari , J. Controlled Release 2023, 358, 667. 10.1016/j.jconrel.2023.05.022 37207794

[213] B. e. Nie , S. Huo , X. Qu , J. Guo , X. Liu , Q. Hong , Y. Wang , J. Yang , B. Yue , Bioact. Mater. 2022, 16, 134. 35386313 10.1016/j.bioactmat.2022.02.003 PMC8958424

[214] P. Feng , R. Zhao , W. Tang , F. Yang , H. Tian , S. Peng , H. Pan , C. Shuai , Adv. Funct. Mater. 2023, 33, 2214726.

[215] G. Lu , Y. Xu , Q. Liu , M. Chen , H. Sun , P. Wang , X. Li , Y. Wang , X. Li , X. Hui , E. Luo , J. Liu , Q. Jiang , J. Liang , Y. Fan , Y. Sun , X. Zhang , Nat. Commun. 2022, 13, 2499. 35523800 10.1038/s41467-022-30243-5 PMC9076642

[216] A. Zheng , X. Wang , X. Xin , L. Peng , T. Su , L. Cao , X. Jiang , Bioact. Mater. 2023, 21, 403. 36185741 10.1016/j.bioactmat.2022.08.031 PMC9483602

[217] S. Khattak , I. Ullah , H. Xie , X.‐D. Tao , H.‐T. Xu , J. Shen , Coord. Chem. Rev. 2024, 509, 215790.

[218] H. Lu , Z. Li , Z. Duan , Y. Liao , K. Liu , Y. Zhang , L. Fan , T. Xu , D. Yang , S. Wang , Y. Fu , H. Xiang , Y. Chen , G. Li , Adv. Mater. 2024, 36, 2408016. 10.1002/adma.202408016 39165073

[219] J. Yao , Q. He , X. Zheng , S. Shen , J. Hui , D. Fan , Adv. Funct. Mater. 2024, 34, 2315217.

[220] W. Zhang , L. Li , Z. Wang , Y. Nie , Y. Yang , C. Li , Y. Zhang , Y. Jiang , Y. Kou , W. Zhang , Y. Lai , Biomaterials 2025, 315, 122959. 39612764 10.1016/j.biomaterials.2024.122959

[221] H. Huang , L. Qiang , M. Fan , Y. Liu , A. Yang , D. Chang , J. Li , T. Sun , Y. Wang , R. Guo , H. Zhuang , X. Li , T. Guo , J. Wang , H. Tan , P. Zheng , J. Weng , Bioact. Mater. 2024, 31, 18. 37593495 10.1016/j.bioactmat.2023.07.004 PMC10432151

[222] M. Nguyen , M. Karkanitsa , K. L. Christman , Nat. Rev. Bioeng. 2024, 2, 810.

[223] X. Xue , Y. Hu , Y. Deng , J. Su , Adv. Funct. Mater. 2021, 31, 2009432.

[224] M. Mirkhalaf , Y. Men , R. Wang , Y. No , H. Zreiqat , Acta Biomater. 2023, 156, 110. 35429670 10.1016/j.actbio.2022.04.014

[225] Y. Zhang , J. Xu , Z. Fei , H. Dai , Q. Fan , Q. Yang , Y. Chen , B. Wang , C. Wang , Adv. Mater. 2021, 33, 2106768. 10.1002/adma.202106768 34601760

[226] A. Joshi , S. Choudhury , S. B. Gugulothu , S. S. Visweswariah , K. Chatterjee , Biomacromolecules 2022, 23, 2730. 35696326 10.1021/acs.biomac.2c00423

[227] H. Zhang , N. Montesdeoca , D. Tang , G. Liang , M. Cui , C. Xu , L.‐M. Servos , T. Bing , Z. Papadopoulos , M. Shen , H. Xiao , Y. Yu , J. Karges , Nat. Commun. 2024, 15, 9405. 39477929 10.1038/s41467-024-53646-y PMC11526146

[228] M. Pierrevelcin , V. Flacher , C. G. Mueller , R. Vauchelles , E. Guerin , B. Lhermitte , E. Pencreach , A. Reisch , Q. Muller , L. Doumard , W. Boufenghour , A. S. Klymchenko , S. Foppolo , C. Nazon , N. Weingertner , S. Martin , C. Briandet , V. Laithier , A. Di Marco , L. Bund , A. Obrecht , P. Villa , M. Dontenwill , N. Entz‐Werlé , Adv. Healthcare Mater. 2022, 11, 2200195. 10.1002/adhm.202200195 36057996

[229] E. C. González Díaz , A. G. Lee , L. C. Sayles , C. Feria , E. A Sweet‐Cordero , F. Yang , Adv. Healthcare Mater. 2022, 11, 2200768. 10.1002/adhm.202200768 PMC10162498 35767377

